# The Tumor Suppressor BCL7B Functions in the Wnt Signaling Pathway

**DOI:** 10.1371/journal.pgen.1004921

**Published:** 2015-01-08

**Authors:** Tomoko Uehara, Eriko Kage-Nakadai, Sawako Yoshina, Rieko Imae, Shohei Mitani

**Affiliations:** 1 Department of Physiology, Tokyo Women's Medical University School of Medicine, Tokyo, Japan; 2 Tokyo Women's Medical University Institute for Integrated Medical Sciences, Tokyo, Japan; University of California San Francisco, United States of America

## Abstract

Human *BCL7* gene family consists of *BCL7A*, *BCL7B*, and *BCL7C*. A number of clinical studies have reported that *BCL7* family is involved in cancer incidence, progression, and development. Among them, *BCL7B*, located on chromosome *7q11.23*, is one of the deleted genes in patients with Williams-Beuren syndrome. Although several studies have suggested that malignant diseases occurring in patients with Williams-Beuren syndrome are associated with aberrations in *BCL7B*, little is known regarding the function of this gene at the cellular level. In this study, we focused on *bcl-7*, which is the only homolog of *BCL7* gene family in *Caenorhabditis elegans*, and analyzed *bcl-7* deletion mutants. As a result, we found that *bcl-7* is required for the asymmetric differentiation of epithelial seam cells, which have self-renewal properties as stem cells and divide asymmetrically through the WNT pathway. Distal tip cell development, which is regulated by the WNT pathway in *Caenorhabditis elegans*, was also affected in *bcl-7*-knockout mutants. Interestingly, *bcl-7* mutants exhibited nuclear enlargement, reminiscent of the anaplastic features of malignant cells. Furthermore, in KATOIII human gastric cancer cells, *BCL7B* knockdown induced nuclear enlargement, promoted the multinuclei phenotype and suppressed cell death. In addition, this study showed that BCL7B negatively regulates the Wnt-signaling pathway and positively regulates the apoptotic pathway. Taken together, our data indicate that BCL7B/BCL-7 has some roles in maintaining the structure of nuclei and is involved in the modulation of multiple pathways, including Wnt and apoptosis. This study may implicate a risk of malignancies with *BCL7B*-deficiency, such as Williams-Beuren syndrome.

## Author Summary

*BCL7B*, a member of the human *BCL7* gene family, is deleted in patients with Williams-Beuren syndrome. Although several clinical studies have suggested that malignant diseases occurring in patients with Williams-Beuren syndrome are associated with aberrations in *BCL7B*, little is known regarding the physiological function of this gene. Here, we show that *bcl-7*, the only homolog of *BCL7* gene family in *Caenorhabditis elegans*, regulates asymmetric cell differentiation in somatic “stem-like” seam cells through at least the Wnt pathway and promotes the apoptotic pathway. In addition, *bcl-7* deletion mutants show enlarged nuclei in epidermis and germ cells. Furthermore, in KATOIII human gastric cancer cells, *BCL7B* knockdown induces nuclear enlargement, as observed in *Caenorhabditis elegans*, and promotes the multinucleated phenotype, both of which are reminiscent of malignant diseases. *BCL7B* also negatively regulates the Wnt-signaling pathway and positively regulates the apoptotic pathway, similar to *Caenorhabditis elegans*. Altogether, this study may open the door for understanding the function of BCL7 family in cell differentiation and malignancies.

## Introduction

Cytogenetic abnormalities of chromosome 7 occur frequently in patients with cancer. Patients with certain types of malignant transformation, such as acute lymphoblastic leukemia, myelodysplastic syndrome, or juvenile myelomonocytic leukemia, frequently have deletions or abnormalities in chromosome 7 [1]–[4]. Some of the genes located on chromosome 7 are thought to act as tumor-related genes, with roles in cancer initiation and/or progression. However, few studies have investigated the molecular mechanisms controlled by specific genes located on chromosome 7.

One of the most well-known diseases related to chromosome 7 microdeletions is Williams-Beuren syndrome (WBS), a contiguous gene syndrome with a dominant autosomal inheritance pattern. WBS patients show a variety of phenotypes, including elfin face, mental retardation, reduced spatial reasoning capacity, supravalvular aortic stenosis, and peripheral pulmonic stenosis. In the past three decades, several reports have described the occurrence of malignant diseases in WBS patients [5]–[9]. These reports have shown that patients with WBS are at an increased risk of malignant transformation due to aberrations in candidate genes, such as *BCL7B*.

*BCL7B* is a member of the *BCL7* gene family; members of this gene family, including *BCL7A* and *BCL7C*, located on chromosomes 12 and 16, respectively, have a conserved amino-terminal region as their functional domain [10]. A number of studies have found that *BCL7* family members are involved in cancer initiation, progression, and development. For example, decreased expression of *BCL7A* may be a risk factor for astrocytoma [11], Burkitt lymphoma [12], non-Hodgkin's lymphoma [13], mycosis fungoides [14], and cutaneous T cell lymphoma [15]. Although the *BCL7* gene family is thought to have tumor-associated functions, little is known regarding the specific functional roles of *BCL7* genes; this may be attributed to the functional redundancy among *BCL7* family members, which makes it difficult to analyze the individual roles of *BCL7* genes.

In this study, the functional significance of *C28H8.1* (designated here as *bcl-7*), which shares 41% homology with the amino-terminal region of human *BCL7* gene family and is the only homolog in *Caenorhabditis elegans* (S1A Fig.), was analyzed in the Wnt-signaling pathway and apoptotic pathway. In addition, we also analyzed the function of the *BCL7B* gene in both pathways in KATOIII cells, a human gastric cancer cell line [16].

## Results

### *bcl-7* is required for normal seam cell development in *C. elegans*

First, we knocked down *bcl-7* expression using the feeding RNA interference (RNAi) technique with a *bcl-7*-specific RNAi clone and observed the phenotypes associated with *bcl-7* downregulation in wild-type *C. elegans* hermaphrodites. Downregulation of *bcl-7* in wild-type worms resulted in the egg-laying defective (Egl) phenotype (S1B and S1E Fig.), the protruding vulva (Pvl) phenotype (S1C Fig.), and the burst phenotype (S1D Fig.), reminiscent of defects in epidermal barrier formation [17]. Therefore, we hypothesized that *bcl-7* is involved in the development of the epidermis.

**Figure 1:**
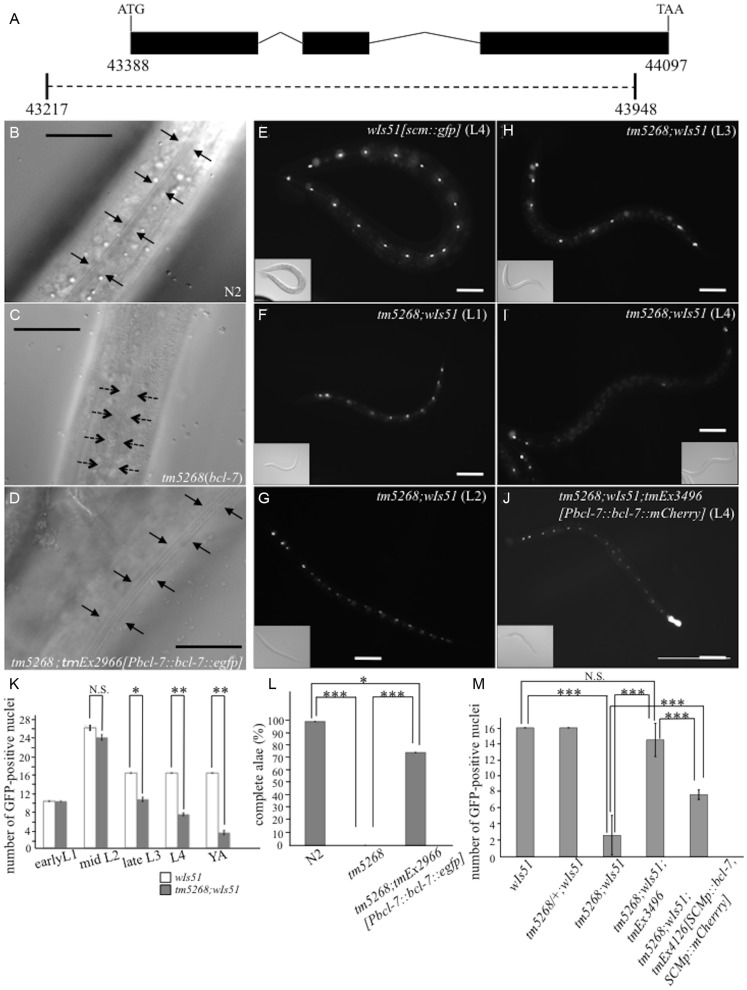
Knockout of *bcl-7* inhibits normal seam cell development in *Caenorhabditis elegans.* **A**: Structure of *bcl-7*. Black boxes, exons; bent lines, introns. The region deleted in the strain *tm5268* is shown as a dotted line, below. The numbers indicate the location in the cosmid *C28H8*. **B–D**: Nomarski images of adult hermaphrodites. A wild-type hermaphrodite had complete alae (the region between arrows) (**B**); Alae were incomplete in *bcl-7* (*tm5268*) hermaphrodites. A region between dotted arrows indicates partial alae with only two ridges. The area without arrows indicates regions without alae (**C**); a transgenic rescue line carrying *Pbcl-7::bcl-7::egfp* reporters (*tm5268;tmEx2966*) with complete alae (three ridges) (**D**). **E–J**: Examples of SCM::GFP localization in wild-type, *tm5268*, and *tm5268;tmEx3496* hermaphrodites with *wIs51* (SCM::GFP). *tm5268;tmEx3496* is a transgenic rescue line carrying *Pbcl-7::bcl-7::mCherry* reporters. A wild-type L4 hermaphrodite expressing SCM::GFP in 16 seam cell nuclei (**E**), *tm5268* hermaphrodites carrying *scm::gfp* reporters (**H**–**G**), and a *tm5268;tmEx3496* hermaphrodite carrying *scm::gfp* reporters (**J**). Inserts show the Nomarski images of the same animals of the fluorescence images. **K**: Bar chart showing the average number of seam cells (about the worms at each larval stage, except for the middle L2 stage, and the young adult stage) or daughter cells (about the worms at middle L2 stage) expressing GFP in wild-type and *tm5268* hermaphrodites (n = 21–46). **L**: Percentages of worms with complete alae in wild-type, *tm5268*, and *tm5268;tmEx2966* adult hermaphrodites (n = 20–30). **M**: Bar chart showing the average number of seam cells expressing GFP in wild-type, *tm5268*, *tm5268;tmEx3496*, and *tm5268;tmEx4126[SCMp::bcl-7, SCMp::mCherry]* adult hermaphrodites with *wIs51* (SCM::GFP) (n = 15–23). Error bars indicate the standard error of the mean (SEM). Asterisks indicate statistical significance compared with each other. *p<0.05. **p<0.005. ***p<0.001. N.S.: no significance. Scale bar  = 25 µm.

Next, to examine the phenotypes produced by *bcl-7* knockout, we generated a *bcl-7* deletion mutant, *tm5268*, containing a deletion of 0.7 kbp (Fig. 1A). Because the deletion covered almost all *bcl-7* regions, including the amino-terminal domain, which is conserved and considered to be the functional domain [10], *tm5268* is practically a null mutant. The *tm5268* mutant showed a variety of phenotypes, including Pvl (the rate was 61.4%; S1F–I Fig.), the alae morphological variant (Fig. 1B–D, and 1L), and sterility (Ste), which suggest the phenotypes in deletion mutants not only reproduced the RNAi experiments but also indicated additional phenotypes. While the *bcl-7* mutants had a normal number of vulval precursor cells at the larval stages, they showed Pvl phenotypes after young adult stages (S1G–H Fig.). In addition, the Pvl phenotype was also observed in *bcl-7* heterozygotes at a rate of 14.3% (S1I Fig.). This result suggests that the phenotype of *bcl-7* deletion mutants is semi-dominant similar to the phenotype of *BCL7B* deletion in human disease, such as Williams-Beuren syndrome. Furthermore, alae, the cuticle structures considered a hallmark of normal seam cell differentiation, were “incomplete” (alae with only one or two ridges) or absent in *tm5268* worms in contrast to wild-type worms (Fig. 1B and 1C). The Pvl phenotype and alae malformation are caused by defects in epidermal cells, particularly epidermal stem-like seam cells [17], [18]. The presence of these phenotypes in *bcl-7* deletion mutants suggests that BCL-7 influences the development of seam cells, which have both self-renewal potential and differentiation capability, in *C. elegans*.

Then, we investigated whether the number of seam cells was altered in *bcl-7* deletion mutants by analyzing transgenic worms carrying the *scm::gfp* transgene [19] as a marker of seam cell nuclei. In a wild-type L4-stage hermaphrodite, there were 16 seam cells on each side (Fig. 1E). By contrast, the number of seam cells was significantly reduced in the mutant worms (Fig. 1I), and *scm::gfp*-negative cells were observed more often in the V cell lineage than in the H and T cell lineages (Fig. 1I). Differences between wild-type worms and *tm5268* worms were found in most larval stages, except for the early L1 stage (Fig. 1F–I and 1K). The expression pattern of another seam cell marker, *cdh-3::gfp* [20], which is localized to the cytoplasm of seam cells, also revealed that the number of GFP-positive cells was lower in *bcl-7* mutants (S2A–D Fig.). Both the defect of alae and the decreased seam cell number were rescued by the introduction of *bcl-7* genomic DNA, *Pbcl-7::bcl-7::egfp* (*tm5268;tmEx2966*) or *Pbcl-7::bcl-7::mCherry* (*tm5268;tmEx3496*) (Fig. 1D and 1J–M). The expression of the rescue constructs was ubiquitous, including in the hypodermis, from the embryonic stage to the adult stage, and BCL-7 was localized to the nuclei (S3A–H Fig.). Therefore, we hypothesized that BCL-7 functions cell-autonomously in seam cells. To test this hypothesis, we analyzed whether expressing a seam cell-specific construct rescues decreased seam cell number. The number of seam cells was significantly increased by the introduction of *bcl-7* genomic DNA under a seam cell-specific promoter (*tmEx4126[scmp::bcl-7, scmp::mCherry]*) (Fig. 1M). These results suggest that BCL-7 is involved in the normal development of seam cells and functions cell-autonomously in seam cells.

Next, we addressed whether the observed decrease in seam cells in *tm5268* worms resulted from the hyperactivation of apoptosis. To examine this, we analyzed whether the apoptotic pathway was hyperactivated in *bcl-7* deletion mutant worms using worms with a mutation in the *ced-3* gene, which encodes a member of the caspase family required for the execution of apoptosis in *C. elegans* [21]. However, *bcl-7*(III)*;ced-3*(IV) double mutants did not exhibit increased numbers of seam cells compared with *bcl-7* single mutants (the average seam cell number in adult, double-mutant hermaphrodites was 3.6 (n = 12)), suggesting that the decrease in the number of seam cells in *bcl-7-*deficient worms is not caused by hyperactivation of apoptosis.

**Figure 2:**
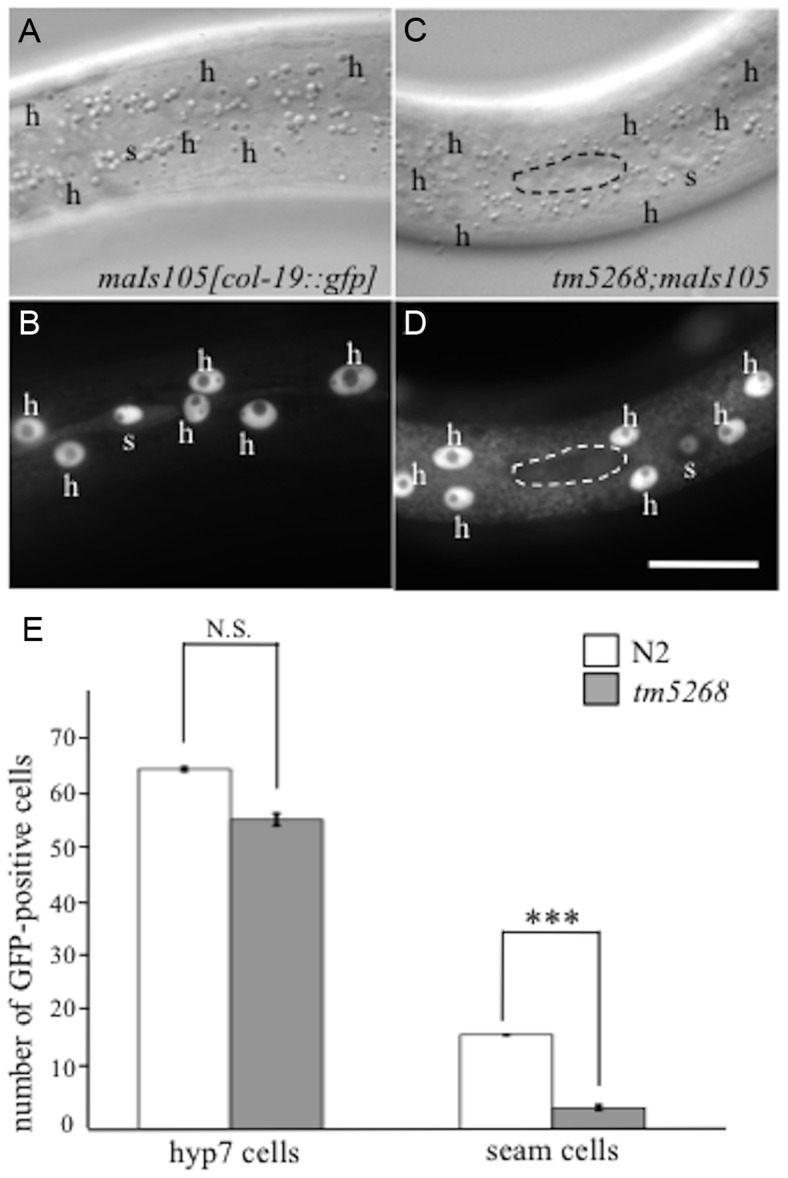
BCL-7 is involved in asymmetric cell differentiation of the epidermis in *Caenorhabditis elegans*. **A–D**: Examples of *col-19p::gfp* localization in wild-type and tm5268 adult hermaphrodites. Nomarski (**A, C**) and GFP images (**B, D**) of wild-type (A, B) and *tm5268* (C, D) adult hermaphrodites carrying *col-19::gfp* reporters. A wild-type hermaphrodite expressing *col-19p::gfp* in both hyp7 cells (‘h’) and seam cells (‘s’), (**B**) and a *tm5268* hermaphrodite expressing *col-19p::gfp* in hyp7 cells but not in seam cells (surrounded with a dotted oval) (**D**). **E**: Bar chart of the cell numbers expressing *col-19::gfp* in wild-type and *tm5268* adult hermaphrodites (n = 15–20) for hyp7 and seam cells. Error bars indicate SEM. Asterisks indicate statistical significance compared with each other. ***p<0.001. N.S.: no significance. Scale bar  = 25 µm.

In wild-type *C. elegans*, seam cells divide asymmetrically during each larval stage. The anterior daughter cell loses its seam cell properties and differentiates into a hyp7 cell, whereas the posterior cell keeps its self-renewal potential, remaining a seam cell (S4S Fig.) [22], [23]. To examine whether the reduction in seam cell numbers in *tm5268* is followed by an increase in the hyp7 cell number, we used transgenic worms that expressed an adult-specific hypodermal marker, *col-19::gfp* [24], [25]. In wild-type animals, *col-19::gfp* was expressed in the hypodermal syncytial hyp7 cells and 16 seam cells (Fig. 2A, 2B and 2E). The number of GFP-positive hyp7 cells in *tm5268* was not increased but tended to decrease compared with the number in the wild-type worms. Additionally, the number of GFP-positive seam cells in *tm5268* was significantly decreased compared with the number in the wild-type worms (Fig. 2C–E). Interestingly, the nuclei of hyp7 cells from *bcl-7* mutants were significantly enlarged and had an irregular shape compared to those from wild-type animals (7.66±0.10 µm and 6.77±0.14 µm, p<0.001; S5A–D Fig.).

To determine whether the somatic stem-like cells acquire another fate in the cell lineage, we analyzed neural cells, including sensory PVD neurons, PDE neurons, and phasmids, derived from V- and T-cell lineages. PVD and PDE neurons originate from an asymmetric division of the V5 cell (S4S Fig.) [23], whereas phasmid cells are generated by the asymmetric T-cell division when the posterior daughter cell maintains the seam cell phenotype and the anterior daughter cell commits to the neural fate, giving rise to the phasmid (S4T Fig.) [23]. We analyzed the cell fates of PVD and PDE in transgenic worms carrying the PVD and PDE markers *des-2::gfp* and *dat-1p::gfp*, respectively [26], [27]. No extra PVD or PDE cells were found in any of the *bcl-7* mutants (n  =  10–15, S4A–J Fig.). In wild-type worms, phasmids can be detected by the uptake of fluorescent dye [28]. Similarly, in all *bcl-7* mutants, both socket cells in the phasmid, but not other cells, absorbed the fluorescent dye in dye-filling assays (n = 10, S4K–N Fig.). These results suggest that stem-like cells fail to acquire a terminal differentiation fate in *bcl-7* mutants.

Next, we hypothesized that more undifferentiated cells are present in *bcl-7* deletion mutants because we observed an increase in the number of cells with enlarged nuclei, as well as the loss of differentiated-cell markers. To test this hypothesis, we analyzed the expression patterns of an undifferentiated state marker, *egl-27/Mta* [29], in wild type and *tm5268* worms carrying the *egl-27p::his-24::mCherry* transgene. The expression pattern of *egl-27* was different between wild type and *bcl-7* mutant worms. In wild type worms, *egl-27* was strongly expressed in the nuclei of intestinal cells (S6A–B Fig.) and weakly expressed in the nuclei of epidermis (S6C–D Fig.) during the L4 stage (n = 10). By contrast, *egl-27* was strongly expressed ubiquitously particularly in the epidermal cells including both seam cells and hyp7 cells in *bcl-7* mutant worms (n = 10, S6E–H Fig.). Furthermore, we analyzed the expression levels of undifferentiated cell markers using a quantitative real-time polymerase chain reaction (qRT-PCR) analysis. Both *egl-27* and *ceh-6*, markers of the undifferentiated state in *C. elegans,* were significantly increased in the *tm5268* mutants compared with the wild type worms (S6I–J Fig.). These results suggest that stem-like cells fail to acquire a terminal differentiation fate in *bcl-7* mutants.

### Loss of BCL-7 function affects gonadal size and germ cell differentiation

**Figure 3:**
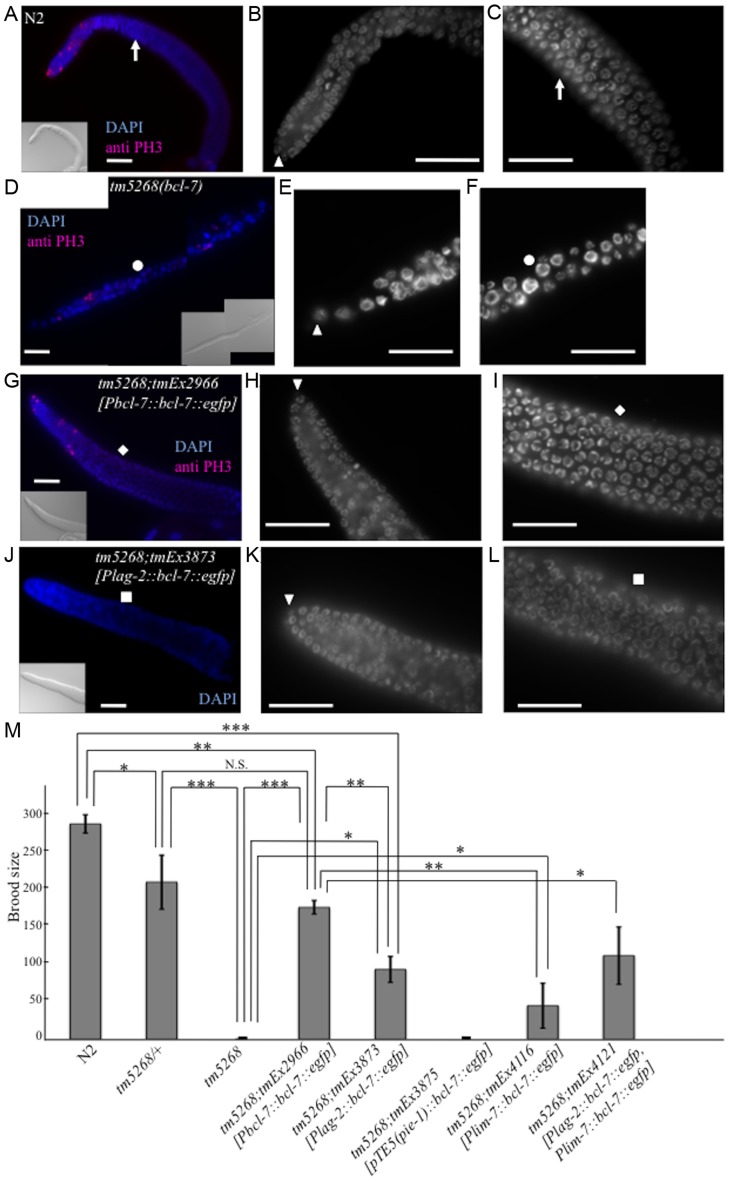
Knockout of *bcl-7* affects gonadal development and germ cell proliferation in *Caenorhabditis elegans*. **A–C**: A dissected adult germline of a wild-type adult hermaphrodite. A dissected gonad stained with DAPI (blue) and an anti-PH3 antibody (pink) as a mitotic cell-specific marker (**A**), and the regions of mitosis (**B**) and meiosis (**C**) of the gonad stained with DAPI. The arrowhead indicates a distal tip cell (DTC). The white arrows indicate the corresponding position of the gonad. **D–F**: A dissected germline of a *tm5268* adult hermaphrodite. A dissected gonad stained with DAPI (blue) and an anti-PH3 antibody (pink) as a mitotic cell-specific marker (D), and the regions of mitosis (**E**) and meiosis (**F**) of the gonad stained with DAPI. The arrowhead indicates a DTC. The white circles indicate the corresponding position of the gonad. **G–I**: A dissected germline of a *tm5268;tmEx2966* adult hermaphrodite. A dissected gonad stained with DAPI (blue) and an anti-PH3 antibody (pink) as a mitotic cell-specific marker (G), and the regions of mitosis (**H**) and meiosis (**I**) of the gonad stained with DAPI. The arrowhead indicates a DTC. The white rhomboid indicates the corresponding position of the gonad. **J–L**: A dissected germline of a *tm5268;tmEx3873* adult hermaphrodite carrying *Plag-2::bcl-7::egfp* as a DTC-specific rescue construct. A dissected gonad stained with DAPI (blue) (**J**), and the regions of mitosis (**K**) and meiosis (**L**) of the gonad stained with DAPI. The arrowhead indicates a DTC. The white squares indicate the corresponding position of the gonad. **M**: Bar chart indicating the brood size in wild-type, heterozygous and homozygous *bcl-7* mutants, and transgenic rescue lines. The extrachromosomal array *tmEx3875* contains *pTE5::bcl-7::egfp* as a germ cell-specific rescue construct, *tmEx4116* contains *lim-7::bcl-7::egfp* as a gonadal sheath cell-specific rescue construct, and *tmEx4121* contains both *Plag-2::bcl-7::egfp* and *lim-7::bcl-7::egfp*. Error bars indicate SEM. Asterisks indicate statistical significance. *p<0.05, **p<0.005, ***p<0.001. N.S.: no significance. Scale bar  = 25 µm.

The Ste phenotype was observed in *bcl-7* deletion mutants (Fig. 3M); specifically, no oocytes were detected in the homozygous mutants, and their gonads were shortened (Fig. 3D). In addition, the brood size of *bcl-7* heterozygotes was significantly reduced compared with that of wild-type worms (Fig. 3M), which indicates that the genetic trait of *bcl-7* mutation in *C. elegans* is haploinsufficiency, similar to that of human diseases. These results revealed that the loss of BCL-7 function affects not only seam cells but also the development of somatic gonads and/or germ cells. To further investigate the gonadal and germ cell phenotypes in *tm5268* worms, we performed diamidinophenylindol (DAPI)-staining and fluorescence immunostaining with an anti-phospho-histone H3 (PH3) antibody as a mitotic marker [30]. In wild-type *C. elegans* hermaphrodites, the mitotic region covered approximately 10–15 cell diameters as previously reported (Fig. 3A) [31]. In *bcl-7* mutant worms, PH3-positive cells were found, but tended to decrease compared with the wild type worms (Fig. 3D). In addition, they were occasionally observed farther from the distal tip cells (DTCs) than in wild type worms. These results suggest that the shortened gonad observed in *tm5268* mutants was not because of the absence of mitosis but rather because of the decrease of mitosis and may be due to the defects of cell differentiation after mitosis. In addition, the average length of the major axis of germ cell nuclei was 3.17±0.03 µm in wild type worms (Fig. 3A–C, S7A Fig.). By contrast, *bcl-7* mutants exhibited irregularly shaped and significantly larger germ cell nuclei measuring 4.42±0.08 µm (Fig. 3D–F, S7B Fig.). Furthermore, the number of germ cells in *tm5268* worms was decreased compared with that in wild-type worms (Fig. 3D–F). Thus, BCL-7 is necessary for gonadal development, particularly for gonadal arm elongation, and for germ cell entry into meiosis. The Ste phenotype, shortened gonads, and enlarged germ cell nuclei in *tm5268* worms were rescued by the introduction of *bcl-7* genomic DNA under the *bcl-7* promoter (Fig. 3G–I, 3M and S7C). The rescue construct was strongly expressed in the nuclei of somatic DTCs and weakly expressed in the nuclei of germ cells and gonadal sheath cells (S3I–L Fig.). These results suggest that the expression of BCL-7 in DTCs, germ cells, and/or gonadal sheath cells is necessary for its function.

To further examine the observed phenotypes, we introduced *bcl-7* genomic DNA under the DTC-specific *lag-2* promoter (*Plag-2::bcl-7::egfp*). The Ste phenotype of *tm5268* worms was partially rescued by the expression of the *bcl-7* gene in DTCs (Fig. 3M). In addition, thin gonads and irregularly shaped nuclei of germ cells were also partially rescued by the expression of this construct (Fig. 3J–L and S7D). These results suggest that BCL-7 expression in DTCs is necessary but insufficient for normal gonadal development. Therefore, we hypothesized that restoring BCL-7 expression in germ cells could rescue the Ste phenotype in *bcl-7* deletion mutants (*tm5268*). To test this hypothesis, we expressed *bcl-7* genomic DNA fused with the promoter, first intron, and 3′-untranslated region (UTR) of the *pie-1* gene, which is expressed exclusively in germ cells (*pTE-5(pie-1)::bcl-7*) [30]. However, the Ste phenotype was not rescued by the introduction of *pTE5*(*pie-1*)*::bcl-7* (*tmEx3875*) (Fig. 3M). Our study showed that BCL-7 was also expressed in gonadal sheath cells (S3I–L Fig., arrows indicate a part of gonadal sheath cells). Gonadal sheath cells play an important role in embryonic germline amplification and larval gonadal elongation [32]. Therefore, we examined whether the expression of BCL-7 in gonadal sheath cells is sufficient for normal gonadal development by introducing *bcl-7* genomic DNA under a sheath cell-specific sequence (*lim-7* promoter and first intron; *tmEx4116[Plim-7::bcl-7::egfp]*) [33], [34]. The Ste phenotype of *tm5268* worms was partially rescued (Fig. 3M). In addition, we introduced both the DTC-specific rescue construct and the sheath cell-specific rescue construct (*tmEx4121[Plag-2::bcl-7::egfp, Plim-7::bcl-7::egfp]*) and found that the brood size was larger than with either the DTC-specific rescue or the gonadal sheath cell-specific rescue alone (Fig. 3M). These results suggest that BCL-7 expression in somatic DTCs and gonadal sheath cells is more important than its expression in germ cells for normal gonadal development.

One of the factors that regulates the size of the gonads and the timing of the entry into meiosis is LAG-2, a homolog of the Notch receptor ligand that is secreted by DTCs [35], [36]. We therefore hypothesized that DTC functions, including LAG-2 secretion, may be impaired in *bcl-7* mutants. We analyzed DTCs using worms carrying the *lag-2p::gfp* transgene (*qIs56*) [37]. Two GFP-positive DTCs were detected in 100% (17/17) of wild-type worms (S8A and B Fig.), whereas only 19.5% (25/128) of *bcl-7* mutant worms were positive for GFP in both DTCs. The remaining 80.5% (103/128) of *bcl-7* mutant worms were positive for GFP in only one DTC. Furthermore, 12.5% (16/128) of *bcl-7* mutants showed mispositioning of the DTCs with the expression of *lag-2p::gfp* (S8E–H Fig. and S1 Table). Interestingly, heterozygous *bcl-7* mutants exhibited the same phenotypes; i.e., 30% (6/20) of heterozygous *bcl-7* mutants had expression of *lag-2p::gfp* on only one DTC and 50% (10/20) showed mispositioning of the DTC with GFP expression (S1 Table, middle row). These differences between homozygous and heterozygous mutants further demonstrate that the phenotype is semi-dominant. These results suggest that BCL-7 partially controls the differentiation of DTCs.

Next, we attempted to determine the time course of BCL-7 function using a chromophore-assisted light inactivation (CALI) assay. We created a light-inactivatable BCL-7::KillerRed fusion protein by substituting *egfp* of the rescue construct with *KillerRed*. We generated transgenic worms (*tmEx3878*) that expressed the *bcl-7* promoter-driven *bcl-7::KillerRed* construct (*Pbcl-7::bcl-7::KillerRed*) in the *bcl-7*-deficient background (i.e., *tm5268*). In the absence of green-light illumination, this transgene rescued the Ste phenotype (similar results were observed independently in three transgenic lines). When the animals were illuminated with a green light, the BCL-7 protein fused to KillerRed was inactivated [38], [39], allowing inactivation of BCL-7 function. We exposed *tm5268;tmEx3878* to a green light starting from the comma stage of the embryo, the early larval L1 stage, or the L2 stage to the young adult stage and analyzed the Ste phenotype. All worms exposed to the green light at the L2 stage were fertile; however, approximately 90% (70/79) of worms illuminated at the comma stage and 54.5% (18/33) of worms illuminated at the early L1 stage retained the Ste phenotype (S7E Fig.). In addition, we performed a pulse experiment to identify the most critical stage in development for the function of BCL-7. We exposed the same transgenic worms to a green light during the comma and early L1 stages and found that only 20% of the worms were fertile (S7E Fig.). These results imply that the expression and function of BCL-7 in DTCs begin during the early L1 stage, which corresponds to the timing of asymmetric cell division in somatic DTCs.

### RNAi-based screening for suppressors of the *bcl-7* mutant phenotypes

**Figure 4:**
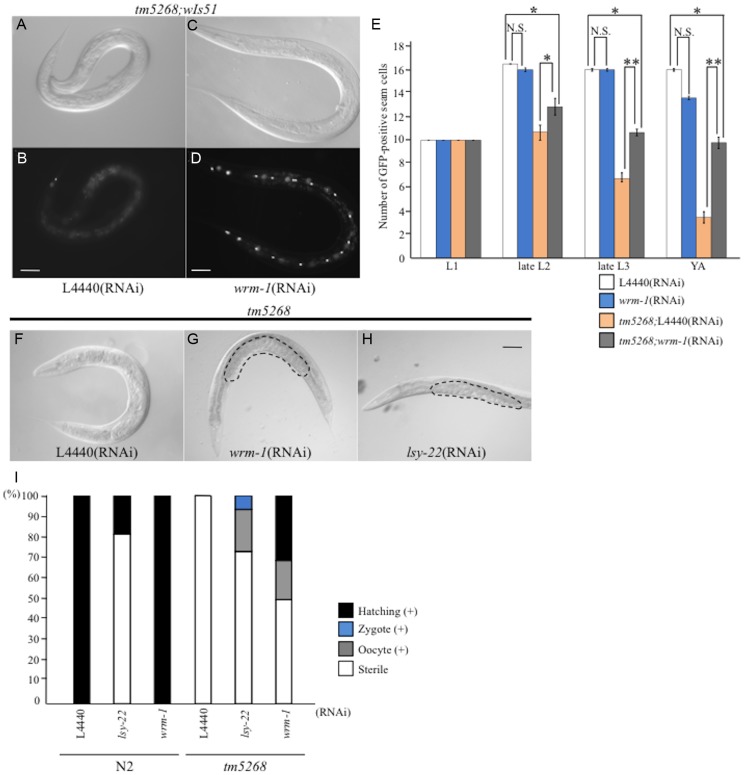
Downregulation of *wrm-1* or *lsy-22* suppresses the phenotypes of *bcl-7* mutants. **A–D**: Nomarski (**A, C**) and GFP images (**B, D**) of *tm5268* adult hermaphrodites carrying an *scm::gfp* reporter treated with empty vector (L4440) (**A, B**) or *wrm-1*-specific RNA (**C, D**). **E**: Bar chart representing the average number of seam cells in wild-type, *tm5268*, *tm5268*;L4440 (RNAi), or *tm5268*;*wrm-1* (RNAi) hermaphrodites at the L1, late L2, late L3, or young adult stages. **F–H**: Nomarski images of *tm5268* treated with L4440 (RNAi), *wrm-1* (RNAi), or *lsy-22* (RNAi). Eggs in the uterus are outlined with dotted lines (**G, H**). **I**: Percentages of the Ste phenotypes in wild-type and *tm5268* adult hermaphrodites treated with L4440 (RNAi), *wrm-1* (RNAi), or *lsy-22* (RNAi). Numbers of examined animals were more than 60 for all strains.Error bars indicate SEM. Asterisks indicate statistical significance. *p<0.05. **p<0.005. N.S.: no significance. Scale bar  = 50 µm.

Loss of BCL-7 function resulted in defects in both seam cells and the gonads, suggesting that BCL-7 controls the asymmetric division of cells in *C. elegans*. More than one genetic pathway is involved in the asymmetric division and differentiation of these cells. Therefore, we next sought to determine the role of BCL-7 in these pathways by screening for genes that could suppress the phenotypes of the *bcl-7* mutant (*tm5268*). Using *bcl-7* mutants, with wild-type worms as the control, we carried out a feeding RNAi screen of 96 genes involved in the development of seam cells and/or gonads, including genes in the WNT/ß-catenin and Notch pathways, heterochronic genes, and other genes that regulate the division and differentiation of seam cells and/or somatic gonadal precursors (SGPs) (functionally classified in S2 Table). We found that downregulation of either *wrm-1* or *lsy-22* suppressed the phenotypes of the *bcl-7* mutant *tm5268* (Fig. 4A–I and S3 Table). Downregulation of WRM-1, a homolog of ß-catenin [40]–[42], suppressed both seam cell reduction and Ste phenotypes. Although LSY-22 is considered a homolog of Groucho-like protein [43] and is also a member of the noncanonical Wnt pathway, *lsy-22*-specific RNAi only partially suppressed the Ste phenotype and did not affect the number of seam cells. Subsequently, we focused on WRM-1, which exhibited potent effects in both somatic cells and germ cells and was expressed in both seam cells and SGPs. Interestingly, the suppressor effect of *wrm-1* RNAi appeared to be dependent on the strength of the RNAi clone (S3 Table). The effect of the *wrm-1c* RNAi clone (constructed with a cDNA generated from wild-type worms) on N2 was stronger than that of *wrm-1* RNAi (from the Ahringer library) and comparable with the phenotypes of the *wrm-1* mutant strains (WormBase; http://www.wormbase.org/). The effect of diluted *wrm-1c* RNAi seemed comparable to RNAi by a genomic clone on suppressing the *bcl-7* mutant's phenotypes (Fig. 4E, S3 Table).

Based on the results of the suppressor screening, we hypothesized that BCL-7 negatively regulates WRM-1 expression. To test this hypothesis, we analyzed the expression levels of *wrm-1* mRNA using qRT-PCR. Additionally, because WRM-1 is one of the three ß-catenin homologs in *C. elegans*, we also analyzed the expression levels of the other ß-catenin homologs, BAR-1 and SYS-1. Compared with wild type worms, *bar-1* and *sys-1* mRNAs were significantly increased in *bcl-7* deletion mutants (S9A Fig.). There was also a trend for increased *wrm-1* in the mutants (S9A Fig.). These results suggest that BCL-7 functions as a negative regulator of the expression of three ß-catenin homologs in *C. elegans*.

**Figure 5:**
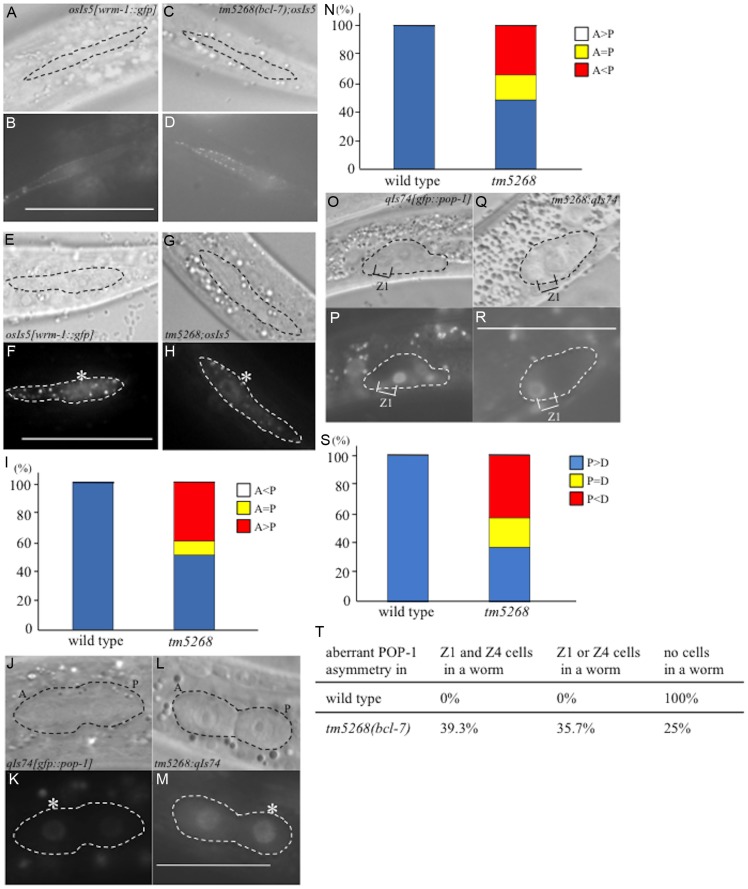
BCL-7 is involved in the asymmetric localization of Wnt components in *Caenorhabditis elegans*. **A–D**: Nomarski (**A, C**) and GFP (**B, D**) images of wild type (**A, B**) and *tm5268* (**C, D**) L2-stage hermaphrodites carrying a *wrm-1::gfp* reporter (*osIs5*) at pre-division states. Seam cells are traced with dotted ovals (**A, C**). **E–H**: Nomarski (**E, G**) and GFP (**F, H**) images showing the localization of WRM-1::GFP in two daughter cells of wild-type or *tm5268* L2 hermaphrodites at post-divisions. The pairs of daughter cells are outlined with dotted ovals. **I**: Frequency of the localization patterns of WRM-1::GFP. The equal and unequal signs indicate the relative intensities of nuclear WRM-1::GFP between two daughter cells. **J–M**: Nomarski (**J, L**) and GFP (**K, M**) images showing the localization of GFP::POP-1 (*qIs74*) in two daughter cells of wild-type (**J, K**) or *tm5268* (**L, M**) L1 hermaphrodites in post-division states. The pairs of daughter cells are outlined with dotted ovals. Asterisks indicate nuclei with stronger expression of GFP. **N**: The frequency of the localization patterns of GFP::POP-1. The equal and unequal signs indicate the relative intensities of nuclear GFP::POP-1 between two daughter cells. **O–R**: Nomarski (**O, Q**) and GFP (**P, R**) images showing the localization of GFP::POP-1 (*qIs74*) in SGPs of wild-type (**O, P**) or *tm5268* (**Q, R**) early L1 hermaphrodites. SGPs are outlined with dotted ovals. **S**: The frequency of the localization patterns of GFP::POP-1. The equal and unequal signs indicate the relative intensities of nuclear GFP::POP-1 between two daughter cells. Anterior is oriented toward the left, and ventral is oriented toward the bottom. **T**: Percentages of worms with the aberrant POP-1 asymmetry in both Z1 and Z4 cells, either Z1 or Z4 cells, and no cells in one worm between wild type and *bcl-7* mutant worms. Numbers of examined samples were more than 30 for all analyses. Scale bar  = 25 µm.

The phenotypes of the *bcl-7* deletion mutants were different from the *pop-1* deletion mutants and *wrm-1*(*gf*) mutants, which are associated with hyperactivation of the Wnt pathway. Therefore, we hypothesized that BCL-7 not only functions as a negative regulator but also affects the Wnt pathway in a different mechanism. Because WRM-1/ß-catenin regulates the levels of POP-1 in the nuclei of target cells during asymmetric cell division, we assumed that the cellular distribution of WRM-1 or POP-1 may be altered in *bcl-7* mutants, and therefore we analyzed the localization of WRM-1 and POP-1 using WRM-1::GFP [44] and GFP::POP-1 [45] fusion reporter proteins. In wild-type worms, the cellular localization of WRM-1::GFP in mother seam cells at the L2 stage was observed near the cell cortex in the anterior half of the cells, as shown by previous reports (Fig. 5A–B) [44]. Furthermore, after division, the fluorescence intensity of WRM-1::GFP was stronger in posterior daughter cells than in anterior cells (Fig. 5E–F). In *bcl-7* mutants, the cellular localization of WRM-1 before cell division was similar to that in wild-type worms (Fig. 5C–D). However, in daughter cells, the fluorescence intensity in the anterior cells was equal to or stronger than that in the posterior cells in approximately 50% of *tm5268* mutant worms (Fig. 5G–I). The asymmetric localization pattern of GFP::POP-1 in *tm5268* worms was also different from that in wild-type worms (Fig. 5J–N), as expected due to the change in WRM-1 localization.

We also analyzed the localization of POP-1 in somatic gonadal precursor Z1 and Z4 cells and their daughter cells using *gfp::pop-1* reporter transgenes. In wild-type worms, GFP expression was stronger in the nuclei of Z1.p and Z4.a cells than in Z1.a and Z4.p cells (Fig. 5O–P). By contrast, approximately 60% of *tm5268* mutants showed aberrant POP-1 localization (Fig. 5Q–S). In addition, 35.7% of the *tm5268* mutants showed both wild type and aberrant POP-1 asymmetry within the same gonad, as shown in Fig. 5T.

Next, we determined whether the impaired nuclear localization of POP-1 disturbs the asymmetric localization of gene products downstream of POP-1 in DTCs. HLH-2 is a downstream factor in the POP-1/TCF pathway [46], [47] and an important transcription factor regulating LAG-2; therefore, it is a determining factor in DTC development. We analyzed the localization of HLH-2 using an *hlh-2p::gfp::hlh-2* reporter construct (*qyIs174*) and found that only 28.2% (13/46) of *bcl-7* mutants showed *hlh-2p::gfp::hlh-2* expression in two DTCs, whereas 100% (10/10) of wild type worms were positive for GFP in two DTCs (S8I–J Fig., and S4 Table). The remaining mutants showed decreased *hlh-2p::gfp::hlh-2* expression, with either no expression observed or expression observed in only one DTC. In addition, 46% (21/46) of the *bcl-7* mutants showed mispositioning of the DTCs (S8K–N Fig. and S4 Table). This result is consistent with the expression pattern of *lag-2p::gfp* in the mutant worms (S8C–H Fig.). HLH-2 is an important transcription factor that regulates LAG-2 and is thus a determining factor for DTCs; therefore, this result may also partially explain the defects in gonadal development in the *bcl-7* mutant (*tm5268*). Taken together, our data suggest that BCL-7 regulates POP-1 distribution in the Z-cell lineage by controlling WRM-1 activity and therefore affects the expression pattern of HLH-2.

### BCL-7 affects the apoptotic pathway in *C. elegans*

BCL-7 has several functions in *C. elegans*. Therefore, we examined whether BCL-7 regulates the apoptotic pathway by analyzing the number of PLM neurons using transgenic worms carrying the *Pmec-4::gfp* transgene (*bzIs8*) [48] as a marker of PLM nuclei. The number of PLM neurons should either decrease or increase if the apoptotic pathway is activated or suppressed, respectively, in *tm5268* mutant worms, because one cell in the PLM lineage undergoes to apoptosis. In wild type worms, two PLM neurons were observed in each worm as shown in S4O–P Fig. (n = 10). Similarly, in *bcl-7* deletion mutants, two PLM neurons were exhibited in the tail of each worm (n = 12, S4Q–R Fig.). This result showed that extra neurons, which are caused by the suppression of the apoptotic pathway, were not found in *bcl-7* mutants. Next, we analyzed whether the levels of apoptosis-related factors were increased in *tm5268* mutant worms. The expression of the anti-apoptotic factor, *ced-9*, was significantly increased in *tm5268* mutant worms compared with wild type worms (S9B Fig.). These results suggest that the apoptotic pathway is suppressed in *tm5268* mutant worms.

### *BCL7B* regulates the size of nuclei in human gastric cancer cells

**Figure 6:**
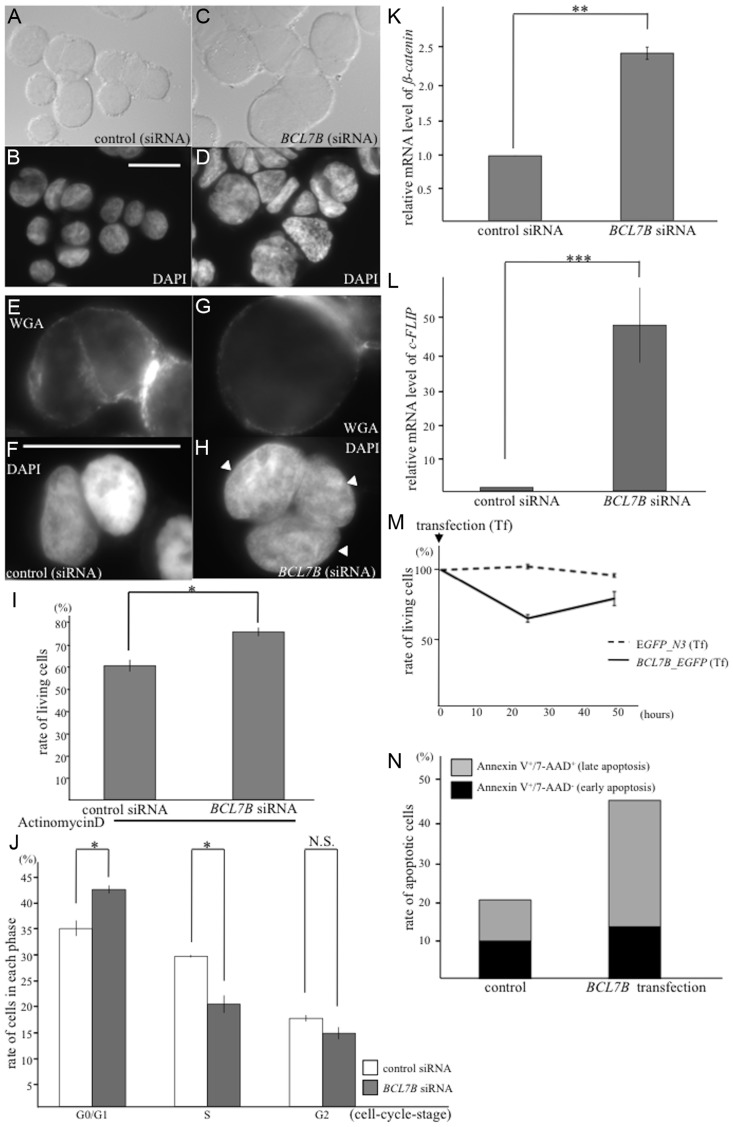
BCL7B functions as a tumor suppressor via multiple pathways. **A–D**: Nomarski (**A, C**) and DAPI (**B, D**) images of KATOIII cells transfected with control siRNA (**A, B**) and *BCL7B*-specific siRNA (**C, D**). **E–H**: Fluorescence images of KATOIII cells stained with WGA-Alexa Fluor 488 (**E, G**) and DAPI (**F, H**). These cells were transfected with control siRNA (**E, F**) or *BCL7B*-specific siRNA (**G, H**). The arrowheads indicate the presence of three nuclei in one cell. **I**: The percentages of the surviving KATOIII cells after transfection with control siRNA or *BCL7B*-specific siRNA and addition of actinomycin D. The number of living cells was counted 5 h after the addition of actinomycin D using trypan blue dye exclusion; this number was then divided by the total cell number. **J**: Percentages of KATOIII cells transfected with control siRNA or *BCL7B*-specific siRNA in the G_0_/G_1_, S, and G_2_ phases. More than twenty thousand cells were counted for each analysis. **K, L**: The expression levels of ß-catenin (**K**) and c-FLIP (**L**) by the qRT-PCR analysis. **M**: Survival rates of KATOIII cells transfected with *EGFP*, as a control, or *BCL7B_EGFP*. The number of living cells was counted using trypan blue dye exclusion, and this number was then divided by the total cell number. **N**: The rates of apoptotic KATOIII cells transfected with *EGFP*, as a control, or *BCL7B_EGFP*. Apoptotic cells were stained for Annexin-V and/or 7-AAD and monitored by flow cytometric analysis. More than ten thousand cells were counted. Each experiment was performed three times independently. Error bars indicate SEM. The asterisks indicate the statistical significance of differences between the two groups. *p<0.05, **p<0.005, ***p<0.001. N.S.: no significance. Scale bar  = 25 µm.

As described above, BCL-7 likely affects the morphology of nuclei and functions in the Wnt-signaling pathway in the development of *C. elegans*. Because *bcl-7* is a homolog of the human *BCL7B* gene, we wondered whether *BCL7B* has similar roles in humans. To examine this, we used KATOIII cells, which are derived from gastric signet-ring cell cancer and express only BCL7B of the BCL7 family members. First, we examined whether siRNA-mediated *BCL7B* knockdown results in nuclear enlargement, as was observed in *C. elegans bcl-7* mutants. As expected, KATOIII cells transfected with *BCL7B* siRNA showed enlarged nuclei compared with control cells transfected with nontargeting siRNA (170.8 ±7.8 µm^2^ and 98.0±4.5 µm^2^; Fig. 6A–D, and S10A–B Fig.). In addition, the occurrence of multinucleated cells (containing two or more nuclei) was increased in *BCL7B*-knockdown cells compared with control cells (the rates of multinucleated cells were 18.8% and 5.7%, respectively; Fig. 6E–H). The average number of nuclei per cell was 1.13 in control cells and 1.41 in *BCL7B*-knockdown cells (P<0.05, Student's *t*-test). In general, cell nuclear enlargement and multinucleated cells are observed in undifferentiated cells, such as cancer cells. Therefore, we hypothesized that the downregulation of *BCL7B* is involved in cell differentiation. To test this hypothesis, we analyzed the expression levels of undifferentiated markers of human cells using qRT-PCR. We found that the stem cell markers, *Nanog*, *Oct3/4*, and *Sox2*, were increased in *BCL7B*-knockdown KATOIII cells (S11A Fig.). This result suggests that BCL7B affects cell differentiation in KATOIII cells, similar to its role in *C. elegans*. Because enlarged nuclei are generally a result of uncontrolled DNA synthesis [49], we next tested whether *BCL7B*-knockdown cells exhibited aneuploidy or cell cycle defects. According to the results of a cell cycle assay, *BCL7B* knockdown did not induce aneuploidy (S10C–D Fig.) but did result in a significant accumulation of cells in the G_0_/G_1_ phase and a decrease in cells in the S phase (Fig. 6J and S10C–E Fig.). Because the observed nuclear enlargement was not induced by an alteration in DNA synthesis, we hypothesized that this phenomenon was caused by increased RNA levels. To test this hypothesis, we analyzed the RNA content of the nucleus by determining the fluorescence intensity of ethidium bromide-stained cells with or without RNase. We found that the fluorescence intensity of ethidium bromide in cells transfected with *BCL7B* siRNA was significantly stronger than in control cells. Furthermore, the addition of RNase eliminated the difference between the control cells and *BCL7B*-knockdown cells (S12A–I Fig.). The expression of *nuclear paraspeckle assembly transcript 1* (*NEAT1*), a noncoding RNA and the core molecule of nuclear paraspeckle [50], was increased in *BCL7B*-knockdown cells compared with control cells (S12J Fig.). These results suggest that BCL7B plays a role in cell cycle progression and in the maintenance of the nuclear structure.

### BCL7B regulates several signaling pathways including Wnt and apoptosis

Next, we examined whether BCL7B is involved in the Wnt signaling pathway in human cells, as observed with BCL-7 in *C. elegans*. We analyzed the expression of *ß-catenin*, an important member of the Wnt signaling pathway, and *high-mobility group A1* (*HMGA1*), one of the target genes of the Wnt pathway [51]. According to a qRT-PCR analysis, the expression levels of *ß-catenin* and *HMGA1* were significantly increased in *BCL7B*-knockdown cells (Fig. 6K, S10F and S10G Fig.). These results suggest that BCL7B functions as a negative regulator of the Wnt pathway in KATOIII cells.

We then analyzed the effects of *BCL7B* overexpression by transfecting KATOIII cells with a *BCL7B_EGFP* fusion construct or *EGFP* alone. Our experiments demonstrated that *BCL7B* overexpression modulated cell proliferation (S13A Fig.). Specifically, 25.5% of *BCL7B*-overexpressing cells died two days after transfection with *BCL7B_EGFP*, compared with 12.3% of control *EGFP* cells (Fig. 6M). Therefore, we hypothesized that overexpression of *BCL7B* may promote apoptosis. To test this hypothesis, we analyzed the number of apoptotic cells using flow cytometry. Indeed, overexpression of *BCL7B* resulted in increased apoptosis compared with the control (Fig. 6N, and S13B–C Fig.). To determine whether the apoptotic pathway was conversely inhibited in *BCL7B*-knockdown cells, we analyzed the survival of *BCL7B*-knockdown cells and control cells following treatment with actinomycin D. The rate of surviving cells was increased in *BCL7B*-knockdown cells compared with control cells (Fig. 6I). We next analyzed the expression of apoptosis inhibitors. qRT-PCR analysis showed that the expression of cellular *FLICE-like inhibitory protein* (*c-FLIP*), which inhibits apoptosis by antagonizing caspase-8 and caspase-10 [52], [53], was significantly upregulated in *BCL7B*-knockdown cells (Fig. 6L). Another apoptosis inhibitor, *Bcl2,* was also significantly increased but the apoptotic factor, *PTEN*, was not increased in *BCL7B*-knockdown KATOIII cells. Interestingly, *Bax*, which binds *Bcl2* and functions as a positive regulator of apoptosis, was increased in *BCL7B*-knockdown KATOIII cells (S11B Fig.). However, the degree of increase in *Bcl2* expression was much larger than the increase in *Bax2* expression; therefore, the *Bcl2*/*Bax* ratio was increased in *BCL7B*-knockdown KATOIII cells, similar to what has been observed in other apoptosis-resistant cells [54]. Collectively, these results suggest that BCL7B is a positive regulator of apoptosis in KATOIII cells and support the hypothesis that BCL7B contributes to the apoptotic pathway.

## Discussion

Malignant diseases are caused by defects in cell differentiation, cell cycle progression, DNA repair mechanisms, or apoptotic pathways [55]–[57]. Although previous studies have revealed the genetic backgrounds of certain types of cancer—for example, the *MSH2* gene in familial nonpolyposis colon cancer [58], the *BRCA1* gene in familial breast cancer [59]–[61], the *APC* gene in hereditary adenomatous polyposis [62], [63], and the *RB* gene in retinoblastoma [64], [65]—the functional mechanisms leading to cancer initiation, progression and development remain to be elucidated. *BCL7B*, located on chromosome 7, is a member of the *BCL7* family of genes and is thought to be a tumor-associated gene [7]–[9]. In this study, we investigated the functional roles of the *bcl-7* and *BCL7B* genes in the Wnt signaling pathway and apoptosis in *C. elegans* and in human gastric cancer cells, respectively. Our results revealed that BCL-7 regulates terminal cell differentiation in somatic “stem-like” seam cells and SGPs via negative regulation of the Wnt pathway in *C. elegans*. BCL-7 also functions as a positive regulator of apoptosis by inhibiting the expression of an anti-apoptotic factor. Additionally, similar to its role in *C. elegans*, human BCL7B functions as a negative regulator of Wnt signaling, presumably upstream of ß-catenin, and induces apoptosis in gastric cancer cells. Furthermore, this study revealed that BCL-7 and BCL7B are also involved in the mechanisms of nuclear enlargement, which is an important signature of malignancy. Collectively, our data suggest that the members of the BCL7 family and their homolog proteins may function as tumor suppressors by affecting multiple pathways (S14 Fig.). Therefore, patients with WBS who have a heterozygous deletion in *BCL7B* may be at risk of malignancy. This study may also be clinically significant in terms of the long-term medical care of WBS patients.

### BCL-7 and BCL7B are involved in the Wnt signaling pathway

Our data demonstrated that *BCL7B* interacts with the Wnt signaling pathway, similar to its *C. elegans* homolog *bcl-7*. The knockdown of *bcl-7* increased the expression of three ß-catenin homologs, *bar-1*, *sys-1*, and *wrm-1*. Therefore, BCL-7 may function as a negative regulator upstream of ß-catenin in the Wnt/ß-catenin pathway in *C. elegans*. In humans, BCL7B is a component of the SWI/SNF complex [66], which has multiple functions, including regulation of the Wnt pathway through transcriptional control of related signaling molecules [67]. In *C. elegans*, the SWI/SNF complex regulates asymmetric cell division and may be associated with Wnt signaling [68], [69]. Although there is currently no evidence of an association between BCL-7 and the SWI/SNF complex in *C. elegans,* our data are consistent with a recent report [68]. Therefore, the SWI/SNF complex may represent the link between BCL-7 and the Wnt pathway.

The Wnt pathway is known to regulate the asymmetric division of most somatic cells in *C. elegans* through the asymmetric localization of Wnt components [70]. Our results showed that *bcl-7* knockout also induced defects in the localization of WRM-1 and POP-1 in the target cell nuclei. Specifically, *bcl-7* deletion caused a reversal of cell polarity and occasionally a loss of cell polarity in seam cells and SGPs. Yamamoto et al. (2011) [71] found that the knockout of multiple Wnt ligands resulted in the randomization, but rarely the loss, of cell polarity. By contrast, knockout of *apr-1*/*APC* resulted in the symmetrical localization of WRM-1 to both the anterior and posterior nuclei by inhibiting the export of WRM-1 from the anterior nuclei [72], [73]. Thus, the functions of BCL-7 and multiple Wnt ligands or APR-1 may be similar. BCL-7 may affect the translocation of WRM-1 from the cortex to the nucleus or the export of WRM-1 from the nucleus. In addition, multiple molecules, including WRM-1 itself, regulate the asymmetric localization of WRM-1. Therefore, BCL-7 may also interact, either directly or indirectly, with WRM-1 itself to regulate its asymmetric localization in the nuclei of target cells and maintain both nuclear WRM-1 and nuclear POP-1 at appropriate levels in *C. elegans*.

As described above, the suppressor screening supported the idea that BCL-7 functions mostly as a negative regulator of the Wnt pathway in *C. elegans*. A weak downregulation of WRM-1 had little effect on wild type worms but was sufficient to partially suppress the phenotype induced by *bcl-7* deficiency (S3 Table). In general, activating the expression of Wnt-related molecules, such as *wrm-1*(*gf*) mutants and *pop-1* mutants, results in an increased number of seam cells, as indicated by downregulation of terminal differentiation markers and upregulation of stem cell markers (Fig. 2, S2 Fig., S6 Fig., S11 Fig.) [74], [75]. However, knockout of BCL-7, which is thought to result in the activation of the Wnt pathway, results in a decreased number of seam cells. This discrepancy may be caused by an additional function of BCL-7 other than its role in the Wnt pathway. For example, BCL-7 can affect seam cell development not only by suppressing the Wnt pathway but also by regulating the terminal cell differentiation of seam cells. The phenotype of fewer seam cells observed in *tm5268* mutants is caused by the disturbance of these two mechanisms.

In this study, we show that BCL-7 suppresses the expression of the *ß-catenin* homologs, *bar-1*, *sys-1*, and *wrm-1*. However, RNAi-based screening showed that only *wrm-1* was a suppressor of the *tm5268* mutant phenotype. This discrepancy may be the result of differences in the degree of BCL-7′s effect on the mRNA expression level of these genes in *tm5268* mutants. The expression of both *bar-1* and *sys-1* is markedly increased in *tm5268* mutant worms (S9A Fig.); therefore, the moderate downregulation of these genes caused by feeding RNAi may not be enough to affect the phenotypes in *bcl-7* deletion mutants. By contrast, the mRNA level of *wrm-1* is only approximately doubled in *tm5268* mutants, therefore, even weak downregulation of *wrm-1* may be sufficient to partially suppress the phenotypes of *tm5268* mutants.

Interestingly, our findings showed that knockdown of *lsy-22* also partially suppressed the Ste phenotype in *bcl-7*-knockout mutants. LSY-22 is a homolog of the Groucho-like protein and is thought to promote expression of the Groucho homolog UNC-37 [43], thereby repressing the transcription of target genes in *C. elegans*. In our experiments, knockdown of *lsy-22* by RNAi partially suppressed the phenotype of the *bcl-7* mutant (*tm5268*). This result is inconsistent with a previous study [43] and suggests that the downregulation of *lsy-22* inhibits the Wnt pathway.

### BCL-7 may affect the differentiation of DTCs and the development of gonads

LAG-2/Notch is secreted from differentiated DTCs and regulates gonadal development. The Wnt signaling pathway is an important regulator of DTC differentiation. In this study, the DTC-specific expression of BCL-7 rescued both the Ste phenotype and the defects in gonadal development observed in *bcl-7* mutant worms. Although *lag-2* temperature-sensitive (*ts*) mutants or *glp-1*(*ts*) mutants exhibited defects in gonadal development, these mutants had only meiotic cells (no mitotic cells) in their gonads and regular germ cell sizes, unlike the *bcl-7* mutant (*tm5268*) [76]–[78]. This result suggests that BCL-7 is involved in the normal cell differentiation of SGPs and subsequent gonadal development in *bcl-7* mutants is affected by the impairment of normal LAG-2 secretion from differentiated DTCs.

Our study found that BCL-7 was also expressed in gonadal sheath cells, as shown in S3I–L Fig. Differentiated gonadal sheath cells are crucial for gonadal elongation, meiotic maturation of germ cells, and embryogenesis [32]. In this study, gonadal sheath cell-specific expression of BCL-7 partially rescued the Ste phenotype of the *bcl-7* deletion mutant. This result suggests that BCL-7 functions not only in DTCs but also in gonadal sheath cells as a regulator of terminal cell differentiation. Furthermore, BCL-7 was also expressed in germ cells, although we did not demonstrate any cell-autonomous functions of BCL-7 in germ cells (Fig. 3M).

Taken together, these data suggest that BCL-7 functions cell-autonomously at least in DTCs and gonadal sheath cells, similar to in seam cells, and its functions are necessary for terminal cell differentiation and normal gonadal development.

### *bcl-7/BCL7B* shares characteristics with other tumor-suppressor genes

In this study, we show that BCL-7 and BCL7B positively regulate the apoptotic pathway and negatively regulate the Wnt signaling pathway. These characteristics are common with certain tumor-suppressor genes. For example, p53, one of the most well-studied tumor suppressors, inhibits cancer initiation and progression through the induction of apoptosis in abnormal cells [79]–[83]. The results of our qRT-PCR analysis revealed that BCL-7 may function as a positive regulator of the apoptotic pathway in *C. elegans* (S9B Fig.). However, there was a discrepancy between the results of qRT-PCR and PLM number analysis, possibly because suppression of apoptosis may occur in the limited cell population or because the remaining cells that are inhibited apoptosis may not undergo terminal differentiation. BCL7B also functions as a positive regulator of apoptosis by repressing the anti-apoptotic factor Bcl2, much stronger than its repression of the pro-apoptotic factor Bax (Fig. 6L and S11A Fig.). The function of BCL-7 in the apoptotic pathway is in some ways similar to the function of p53, which activates the apoptotic pathway of target cells [54], [79]–[83]. In addition, BCL-7 and BCL7B also negatively regulate the Wnt signaling pathway through suppressing the expression of ß-catenin. Downregulation of *apc*, which promotes the degradation of ß-catenin (thereby affecting Wnt signaling), induces hyperactivation of the Wnt pathway and is involved in the development of colorectal cancer [84]. The role of BCL7B in the Wnt pathway is similar to APC. However, there are some differences between BCL7B and other tumor suppressors. For example, the *Rb* gene, which encodes the retinoblastoma (RB) protein, primarily functions to modulate the G_1_/S-phase cell cycle checkpoint [64], [65], [85], whereas *BCL7B* knockdown increased the rate of G_1_ arrest. This dissimilarity is further reflected by the finding that *BCL7B* knockdown does not induce hyperproliferation. Furthermore, the accumulation of cells in the G_0_/G_1_ phase observed in *BCL7B*-knockdown KATOIII cells is similar to the specific profile of quiescent cancer stem cells, which also accumulate in the G0/G1 phase [86]. Collectively, these findings suggest that *BCL7B* is a novel tumor suppressor gene and is required for the terminal cell differentiation.

Downregulation of *BCL7B* in KATOIII cells induced nuclear enlargement, which is considered a hallmark of undifferentiated cells, such as cancer cells, and is associated with the grade of malignancies in neoplastic diseases [49]. This phenotype was similar to the phenotype of the *bcl-7* mutant (*tm5268*) in *C. elegans*. Because the mechanisms mediating nuclear enlargement are not clearly understood, it is difficult to compare the function of BCL7 with the functions of other tumor-related genes. However, we demonstrated that the mRNA expression of specific genes, e.g., *NEAT1*, is significantly increased in *BCL7B*-knockdown cells. Although a direct interaction between nuclear enlargement and an increase in RNA has not been clearly established, a variety of noncoding RNAs have been shown to play various roles in many types of diseases, including cancer. Additionally, the increase in mRNA expression may indicate that transcriptional hyperactivity occurs in *BCL7B*-knockdown cells, potentially through the loosening of chromatin structure [87]–[90]. Although our experiments did not evaluate the chromatin structure of *BCL7B*-knockdown cells, changes in the chromatin structure may explain the observed nuclear alterations in these cells. Furthermore, the mRNA expression levels of undifferentiated markers were significantly increased in both *bcl-7* knockout worms and *BCL7B*-knockdown cells, similar to observations made in some types of malignant cancer cells [91]. Thus, understanding the roles of BCL7B may provide insights into malignant alterations in nuclei.

It should be noted that nuclear changes are not the only alterations that occur in malignant diseases. Poor differentiation, autonomous growth, unlimited proliferation, immortalization, metastatic ability, angiogenic ability, and other phenotypes also contribute significantly to the development of malignancies. Our study demonstrated that BCL7 is involved in mediating nuclear defects, an indicator of poor differentiation and immortalization, but our study did not provide evidence of an association between BCL7 and other malignant phenotypes. Furthermore, *BCL7B*-knockdown cells did not show hyperproliferation compared with control cells, but they did demonstrate phenotypes similar to cancer stem cells [86]. These results suggest that BCL7B activity contributes only to some malignant phenotypes and that cancer initiation may be caused by a combination of aberrations of BCL7 and abnormalities in other tumor-related genes. Therefore, further investigation of the role of BCL7 in malignant transformation and cancer progression and of molecules that associate with BCL7 family proteins is necessary.

## Materials and Methods

### Strains

All strains of *C. elegans* were seeded with *Escherichia coli* OP50. All experiments were performed at 20°C using standard techniques [92]. The wild-type strain Bristol N2 and some transgenic animals (*wIs51*, *maIs105*, *osIs5*, *qIs74*, *arIs51*, *hmIs4*, *baIs4*, *bzIs8*, *stIs10165*, *qIs56* and *qyIs174*) were obtained from the *Caenorhabditis Genetics Center* (CGC, Minneapolis, MN). Strains carrying the following mutations were obtained from the trimethylpsoralen/ultraviolet-mutagenized library, as described previously [93]. Mutated strains were identified by polymerase chain reaction (PCR) amplification with primers spanning the deletion regions: *bcl-7* (*tm5268*) III and *ced-3* (*tm1196*) IV [48]. Strain *tm5268* was backcrossed twice with N2 and balanced with *hT2 [bli-4 (e937) let-? (q782) qIs48]* (I;III). The primers used in this study for nested PCR screening were as follows: *tm5268*_1^st^ round; 5′-TCC GGA TGA GTT GGA TTG TC-3′, 5′-TGT CAT TTC AGC GTC GCG CA-3′; 2^nd^ round; 5′-GCT CCG TCA GAC TCG TAG AT-3′, 5′-AGT GGC TCC ACC TTG ATA GT-3′.

### Constructs and transgenic worms

To determine the expression pattern of the rescue plasmid in the wild-type hermaphrodite, the *bcl-7* genomic sequence, comprising a 0.3-kbp sequence upstream from the ATG initiation codon of *bcl-7,* as well as the full-length *bcl-7* (0.7 kbp), was PCR amplified from N2 genomic DNA using the following primers: bcl-7_sense, 5′-GGT TCC GCG TGG ATC CCA TTT TGA CGC AAG ATT TGA GAG-3′; bcl-7_antisense, 5′-GCT CAC CAT GCG GCC GCA TGG TTG TTT TGA TGT CAT TTC A-3′. The *pFX_egfp* or the *pFX_mCherry* expression vector was digested with *Bam*HI and *Not*I, and the *bcl-7* fragment (*Pbcl-7::bcl-7*) was cloned into the distinct vectors to generate *Pbcl-7::bcl-7::egfp* and *Pbcl-7::bcl-7::mCherry*, respectively [94].

For the seam cell rescue experiment, full-length *bcl-7* (0.7 kbp) was amplified from N2 genomic DNA; the *pPD95.77_Pscm_mCherry* expression vector was digested with *Sma*I and *Not*I, and the full-length *bcl-7* sequence was cloned into the vector to generate *Pscm::bcl-7*.

For the gonadal sheath cell rescue experiment, the first intron of *lim-7* and the full-length *bcl-7* (0.7 kbp) were amplified from N2 genomic DNA using the following primers: lim-7(1^st^ intron)_sense, 5′-TTC TGG TTC CGC GTG GAT CCG TGA GTG TTT TTT TTT TAA TTT G-3′ and lim-7(1^st^ intron)_antisense, 5′-ATT TGC TGA GTA CAT ACG TTC TGA AAA ATG AAA GCT CGA-3′; bcl-7_sense, 5′-TCA TTT TTC AGA ACG TAT GTA CTC AGC AAA TAG ATC TCA-3′ and bcl-7_antisense, 5′-GCT CAC CAT GCG GCC GCT GGT TGT TTT GAT GTC ATT TCA G-3′. The *pFX_egfp* expression vector was digested with *Bam*HI and *Bgl*II, and the *lim-7(1^st^ intron)::bcl-7* fragment was cloned into the vector to generate *lim-7(1^st^ intron)::bcl-7::egfp*.

For the somatic DTC rescue experiment, the 3-kbp sequence upstream from the ATG initiation codon of *lag-2* and the full-length *bcl-7* (0.7 kbp) were amplified using the following primers: Plag-2_sense, 5′-GGT TCC GCG TGG ATC CTC TTA CAG GTT ATA TTA AAT TCT C-3′ and Plag-2_antisense, 5′-GCT GAG TAC ATA AGG CAA ATT TG-3′; bcl-7_sense, 5′-TGC CTT ATG TAC TCA GCA AAT AG-3′, and bcl-7_antisense, 5′-TCA AAA ATA GAG ATC TTG GTT GTT TTG ATG TCA TTT CAG-3′. The *pFX_egfp* expression vector was digested with *Bam*HI and *Bgl*II, and the *Plag-2::bcl-7* fragment was cloned into the vector to generate *Plag-2::bcl-7::egfp*.

For the germ cell rescue experiment, full-length *bcl-7* (0.7 kbp) was amplified from N2 genomic DNA; the *pTE5_egfp* expression vector was digested with *Bam*HI, and the full-length *bcl-7* sequence was cloned into the vector to generate *pTE5::bcl-7::egfp*.

For the chromophore-assisted light inactivation (CALI) of BCL-7, we prepared *Pbcl-7::bcl-7::KillerRed*. To generate this construct, a *bcl-7* genomic sequence, composed of a 0.3-kbp sequence upstream from the ATG initiation codon of *bcl-7* and the full-length *bcl-7* (0.7 kbp), was PCR amplified from N2 genomic DNA using the following primers: bcl-7-KR_sense, 5′-GGT TCC GCG TGG ATC CCA TTT TGA CGC AAG ATT TGA GAG-3′, and bcl-7-KR_antisense, 5′-GAA CAG GGC GGG GCC GCC CTC CAT TTA TGG TTG-3′. The *KillerRed* coding sequence was amplified from the commercially available *pKillerRed-C* vector (Evrogen, Moscow, Russia) using the following primers: KillerRed_sense, 5′-TAA ATG GAG GGC GGC CCC GC-3′, and KillerRed_antisense, 5′-GCT CAC CAT GCG GCC GCT CCT CGT CGC TAC CGA TGG CGC-3′. *Pbcl-7::bcl-7* and *KillerRed* were cloned into the *pFX* vector at the *Bam*HI and *Not*I sites to produce *Pbcl-7::bcl-7::KillerRed*.

To generate the transgenic lines, constructs were injected into worms at 20–100 ng/µL along with *Pmyo-2::DsRed*, *Plin-44::gfp*, or *scmp::mCherry* as an injection marker (100 ng/µL). The transgenic strains constructed for this study were *tmEx2966 [Pbcl-7::bcl-7::egfp, Pmyo-2::DsRed]*, *tmEx3496 [Pbcl-7::bcl-7::mCherry, Plin-44::gfp]*, *tmEx3873 [Plag-2::bcl-7::egfp, Pmyo-2::DsRed]*, *tmEx3875 [pTE5::bcl-7::egfp, Pmyo-2::DsRed]*, *tmEx3878 [Pbcl-7::bcl-7::KillerRed, Plin-44::gfp], tmEx4126 [scmp::bcl-7, scmp::mCherry]*, *tmEx4116 [Plim-7::bcl-7::egfp, Pmyo-2::DsRed]*, and *tmEx4121[Plag-2::bcl-7::egfp, Plim-7::bcl-7::egfp, Pmyo-2::DsRed]*.

The integrated arrays *wIs51*, containing *scm::gfp*, *maIs105*, containing *col-19::gfp*, and *arIs51*, containing *cdh-3::gfp*, were used to assay the numbers of seam cells and hyp7 cells as well as the differentiation of seam cells. The integrated arrays *qIs74*, containing *pop-1::gfp*, and *osIs5*, containing *scm::wrm-1::gfp*, were used to assay the distribution of POP-1 and WRM-1, respectively, in the seam cells. The integrated arrays *hmIs5*, containing *Pdes-2::gfp*, and *baIs4*, containing *Pdat-1::gfp*, were used to assay the formation of PVD and PDE sensory neurons, respectively. The integrated array *bzIs8*, containing *Pmec-4::gfp*, was used to assay the number of PLM neurons. The integrated array *stIs10165*, containing *egl-27p::his-24::mCherry*, was used to analyze the expression pattern of *egl-27* as an undifferentiated marker. The integrated array *qIs56*, containing *Plag-2::gfp*, and *qyIs174*, containing *Phlh-2::gfp::hlh-2*, was used to assay development of DTCs. These transgenic strains were obtained through CGC.

### Microscopy

Differential interference contrast and fluorescence images were obtained using a BX51 microscope equipped with a DP30BW CCD camera (Olympus Optical Co., Ltd, Tokyo, Japan).

### Analysis of seam cells

To characterize seam cell phenotypes, we scored the number of seam cell nuclei and observed alae formation. The numbers of GFP-positive seam cell nuclei were counted at each larval stage (early L1, middle L2, late L3, and L4) and the young adult stage, and staging was assessed by vulval shapes and gonadal morphologies. Alae formation was observed through a Nomarski microscope at the young adult stage.

### Dye-filling assay in *C. elegans*

We performed a dye-filling assay to observe the phenotype of the phasmid in *bcl-7* mutants as described in a previous report [95]. The assay was performed by incubating the wild-type and *bcl-7* (*tm5268*) hermaphrodites in the DiI solution (10 µg/mL DiI in M9 buffer) for 2 h at room temperature. Thereafter, these specimens were observed under a fluorescence microscope as above.

### Self-brood size of *C. elegans*

To determine the average number of progeny produced by each strain, as well as by the transgenic worms, L4 worms were placed on individual NGM plates. Worms were transferred daily until egg laying ceased, and the total number of produced live progeny was then counted.

### DAPI staining and immunostaining in *C. elegans*

Immunostaining of the gonads was performed as described previously [96]. Transgenic or wild-type animals were placed on a subbed slide in 5.0 µL of M9 buffer containing 1 mM levamisole. The heads or tails of these worms were cut off using a 27-gauge needle to extrude their gonads. The dissected gonads were fixed with 1.0% paraformaldehyde in phosphate-buffered saline (PBS) for 10 min and permeabilized with PBS containing 0.1% Triton X-100 for 3 min. Samples were blocked in PBS containing 2.5% bovine serum albumin (BSA) for at least 30 min. Fixed worms were incubated with an anti-PH3 antibody overnight at 4°C. Samples were washed three times with PBS containing 0.5% BSA, incubated with a secondary antibody conjugated to Alexa 594 (Invitrogen, San Diego, CA) for 1 h, and washed at least twice. Then, samples were suspended in 0.1 µg/mL Prolong Gold containing DAPI (Invitrogen) for more than 15 min and observed under a fluorescence microscope.

### Chromophore-Assisted Light Inactivation (CALI) of BCL-7

For CALI of BCL-7, *bcl-7 (tm5268)* mutants expressing *Pbcl-7::bcl-7::KillerRed* were exposed to a light-emitting diode array (572 nm) from the comma-stage, early L1 stage, and L2 stage. At the adult stage, we observed the worms through a microscope and calculated the rates of worms exhibiting the Ste phenotype. In addition, to determine when *bcl-7* function is most critical during development, we also performed the pulse experiments using the CALI method. *bcl-7* (*tm5268*) mutants expressing *Pbcl-7::bcl-7::KillerRed* were exposed to a light-emitting diode array (572 nm) only during the comma and early L1 stages (the exposure period was 6 hrs). The mutants grew in a dark room at all other stages until they became adults. At the adult stage, the percent of worms with Ste phenotypes was calculated.

### RNA interference

RNA interference analyses (RNAi) were performed by feeding animals with dsRNA-producing bacteria as described previously [97]. Briefly, the RNAi clones were transformed into *E. coli* HT115(DE3), and then, approximately 10–20 P0 animals at the early L1 stage were transferred to plates containing RNAi-bacteria grown on 100 µg/mL ampicillin and 1 mmol/L isopropyl-beta-D-thiogalactopyranoside (IPTG).

For the analysis of the phenotypes of *bcl-7*-knockdown worms, the Egl-grade was scored in the F1 generation at the young adult larval stage. More specifically, we observed the worms under a microscope and categorized them as follows: stage 1: 1- to 8-cell stage embryos present in the uterus; stage 2: 16-cell stage to comma-stage embryos present in the uterus; or stage 3: postcomma-stage embryos present in the uterus [98]. We compared the frequencies of *bcl-7*-knockdown and control worms in each category.

For suppressor screening, the numbers of seam cells and of eggs were counted in the F1 generation at the L4 larval stage. We compared *bcl-7* mutants to wild-type worms, and if there was any remission of the mutant phenotype, we identified the corresponding gene as a candidate suppressor gene.

All RNAi clones, except for *sys-1*, *glp-1*, *cki-1*, *bro-1*, *lin-17*, *mig-14*, and *cwn-1*, were taken from the Ahringer RNAi library. The *sys-1*, *glp-1*, *cki-1*, *bro-1*, *lin-17*, *mig-14*, *cwn-1*, and *wrm-1c* RNAi clones were constructed with the cDNAs generated from wild-type worms using the primers listed in S5 Table. These cDNA fragments were cloned into the L4440 (*pPD129.36*) vector.

### Cell culture and transfection

KATOIII cells obtained through ATCC were maintained in Dulbecco's modified Eagle's medium (DMEM) supplemented with 20% fetal bovine serum at 37°C in a humidified 5% CO_2_ incubator. Transfection of 100 nM siRNA (ON-TARGETplus SMARTpool L-017228-00 or ON-TARGET plus siCONTROL non-targeting siRNA; Dharmacon RNAi Technologies, Lafayette, CO) into cells was performed using Lipofectamine 2000 reagent (Invitrogen) according to the manufacturer's protocol.

### Nuclear size assays

KATOIII cells were grown on 2-well chamber slides (Lab-Tek, Campbel, CA) at 1×10^5^ cells/well for approximately 24 h prior to transfection. Forty-eight hours after transfection with *control-siRNA* or *BCL7B-siRNA*, the cells were fixed with 4% paraformaldehyde for 10 min at room temperature and permeabilized with 0.1% Triton X-100 for 3 min. Then, the cells were washed and incubated in 0.1 µg/mL DAPI stain (Life Technology, Carlsbad, CA) overnight at room temperature in the dark. Samples were observed under a fluorescence microscope (with a UV filter), and acquired images were digitally analyzed with ImageJ software (National Institutes of Health, Bethesda, MA).

### Analysis of the number of the nuclei

KATOIII cells were grown on 2 well chamber slides (Lab-Tek) at 1×10^5^ cells/well for approximately 24 h prior to transfection. Forty-eight hours after transfection of *control-siRNA* or *BCL7B-siRNA*, 1 mg/l wheat germ agglutinin (WGA)-Alexa Fluor 488 (Invitrogen) was added to the cells to stain the plasma membrane, which were then incubated for 10 min at 37°C in the dark. Subsequently, the cells were washed twice, fixed with 4% paraformaldehyde for 10 min at room temperature, and permeabilized with 0.1% Triton X-100 for 3 min. Then, the cells were washed and suspended in 0.1 µg/mL DAPI overnight at room temperature in the dark. The samples were then observed under a fluorescence microscope, and the numbers of the cells and the nuclei were counted; the number of nuclei per cell was also calculated for each sample.

### RNA detection assays in KATOIII cells

KATOIII cells were grown on 2 well chamber slides (Lab-Tek) at 1×10^5^ cells/well for approximately 24 h prior to transfection. Forty-eight hours after transfection with *control-siRNA* (slide-a and slide-b) or *BCL7B-siRNA* (slide-a and slide-b), the cells were fixed with 4% paraformaldehyde for 10 min at room temperature and permeabilized with 0.1% Triton X-100 for 3 min. Thereafter, 0.3 mg/mL RNase (Qiagen, Valencia, CA) was added to the cells on slide-a for all samples, while the cells on slide-b were not treated with RNase; all cells were incubated overnight at 4°C. Then, the cells were washed, incubated with the ethidium bromide (Life Technologies, Gaithersburg, MD) for more than 1 h, washed twice, and suspended in 0.1 µg/mL DAPI overnight at room temperature in the dark. Samples were observed under a fluorescence microscope (DAPI: UV filter; ethidium bromide: 594 nm filter). To adjust the focus of the DAPI image, a 25% neutral density (ND) filter was used, and the exposure time was 300 msec at each data acquisition point; for the EtBr image, a 25% ND filter was also used, and the exposure time was 100 msec at each data. Thereafter, acquired images were digitally analyzed with ImageJ software (National Institutes of Health).

### Cell viability assays

To analyze the effects of *BCL7B* knockdown on cell viability, KATOIII cells were transfected with *BCL7B*-siRNA or control-siRNA (day 0) and incubated until day 2 at 37°C. Then, 0.1% actinomycin D was added to the medium, and the cells were incubated for more than 5 h at 37°C. Cells were then collected, supplemented with 0.2% trypan blue, transferred to a plastic disposable counting chamber, and counted with an automated cell counter (TC20, Bio-Rad laboratories, Hercules, CA).

### qRT-PCR analysis

For worms, total RNA was isolated from young adult animals using TRIzol reagent (Invitrogen) according to the manufacturer's instructions. For KATOIII cells, total RNA was extracted using TRIzol (Invitrogen). Total RNA was used for reverse transcription with the Superscript III reverse transcriptase (Invitrogen) using an oligo(dT) primer (for worms) or random primers (for KATOIII cells), according to the manufacturer's instructions, and was subsequently diluted with nuclease-free water (Sigma-Aldrich, St. Louis, MO). qRT-PCR amplification mixtures (25 µL) contained 25 ng of template cDNA, 12.5 µL of 2× SYBR Green I Master Mix buffer (Applied Biosystems, Framingham, MA), and 300 nM of each forward and reverse primers. Reactions were performed on an ABI PRISM 7500 Sequence Detector (Applied Biosystems). The cycling conditions comprised 10 min polymerase activation at 95°C. All data was normalized to *ama-1* gene (for the worms) or *GAPDH* gene (for KATOIII cells). The primer pairs used in this study are listed in S6 Table and S7.

### Cell cycle analysis

After 48 h of BCBL7B-siRNA or control-siRNA transfection, cells were applied to a CycleTEST PLUS DNA Reagent Kit (Becton Dickinson Biosciences, San Diego, CA) according to the manufacturer's instructions. Thereafter, cells were subjected to flow cytometry with a Cell Lab Quanta SC flow cytometer (Beckman-Coulter, Fullerton, CA). For the cell cycle analysis, unstained non-treated samples were used to set the EV gain (set at 0.25) and the FL1 photomultiplier tube (PMT) voltages (set at 3.26). These experiments were repeated three times independently.

### Analysis of apoptotic cells

After 48h of BCBL7B-siRNA or control-siRNA transfection, cells were applied to an Annexin V/PE/7-AAD kit (BD Biosciences) according to the manufacturer's instructions. Thereafter, cells were subjected to flow cytometry. FL1 was used to measure the Annexin V fluorescence, and 7-AAD fluorescence was detected using FL3. Annexin V-positive and 7-AAD-positive cells were considered to be late apoptotic cells, and Annexin V-positive and 7-AAD-negative cells were considered to be early apoptotic cells [99], [100]. The gating lines were drawn based on the viable cells as the negative control, which were Annexin V-negative and 7-AAD-negative cells. Unstained and single-stained samples were used to set the EV gain (at 0.25), FL1 and FL3 PMT-voltages (at 4.08 and 4.50, respectively), and to compensate for Annexin V spillover into the 7-AAD channel. These experiments were repeated three times independently.

### Statistical analyses

All the data were compared using Student's *t*-test.

## Supporting Information

S1 Fig Decrease or loss of BCL-7 results in a variety of phenotypes, including Egl, Pvl, and Burst in *Caenorhabditis elegans*. **A**: A schematic diagram of protein homology of human BCL7 family (hsBCL7A, hsBCL7B, and hsBCL7C), Mus musculus homolog of BCL7B (Mm-Bcl7b), and *C. elegans* homolog (CeBcl-7) adapted from Ref.10. Numbers indicate amino acid positions; gray boxes indicate high homologous sequences; white boxes indicate the regions that are not conserved in *C. elegans*. **B–D**: Nomarski images of adult hermaphrodites treated with *bcl-7*-RNAi. Arrowheads indicate the Pvl phenotype. An arrow indicates the Burst phenotype. **E**: A bar chart representing the frequency of worms with the Egl phenotype (n = 50–70, and these experiments were repeated five times independently). Egg-laying behavior was assayed as follows. The developmental stage of eggs inside the uteri of the worms, determined by microscopy, was categorized as the 1- to 8-cell stage (stage 1; 1); 16-cell stage to precomma stage (stage 2; 2); or comma to postcomma stage (stage 3; 3). **F–H**: Nomarski images of a wild-type (**F**) and *tm5268* hermaphrodites (**G**) at the L4 stage and at the adult-stage (**H**). **I**: Percentages of the Pvl phenotypes in wild type and *tm5268* mutant worms (n>80). Error bars indicate SEM. Asterisks indicate the statistical significance of differences between groups. *p<0.05. ***p<0.001. N.S.: no significance. Scale bar  = 50 µm.
(TIFF)

S2 Fig Knockout of *bcl-7* inhibits normal epidermal development in *Caenorhabditis elegans*. **A–D**: Examples of GFP localization in wild-type and *tm5268* hermaphrodites carrying the *cdh-3::gfp* reporter. Nomarski (**A, C**) and GFP (**B, D**) images of wild-type (**A, B**) and *tm5268* (**C, D**) adult hermaphrodites carrying the *cdh-3::gfp* reporter. A white arrow indicates the absence of a seam cell. Scale bar  = 25 µm.
(TIFF)

S3 Fig BCL-7 is ubiquitously expressed. **A–L**: Nomarski (**A, C, E, G, L, K**) and GFP (**B, D, F, H, J, L**) images of wild-type adult hermaphrodites carrying the *Pbcl-7::bcl-7::egfp* reporter. BCL-7 is expressed in the nuclei of neurons (bracket) (**B**), the seam cells (‘s’) and hyp7 cells (‘h’) (**D**), and intestines (white arrows) (**F**) of worms. BCL-7 is expressed in the early embryonic stage (asterisk) (**H**). BCL-7 was expressed in germ cells and is strongly expressed in a somatic distal tip cell (DTC) (**J, L**). A higher magnification view of the white square is presented in the lower panel of the images (**K, L**). The arrowhead indicates strong GFP expression in the DTC. The white arrows indicate GFP expression in gonadal sheath cells. Scale bar  = 50 µm.
(TIFF)

S4 Fig Knockout of *bcl-7* has no effect on the development of neuronal cells. **A–F**: Expression patterns of DES-2::GFP in wild-type (n = 10) and *tm5268* (n = 12) hermaphrodites carrying the *des-2::gfp* reporter (*hmIs4*). Nomarski (**A, C**) and GFP (**B, D, E, F**) images of wild-type (**A, B, E**) and *tm5268* (**C, D, F**) adult hermaphrodites. Two PVD neurons (white arrows) without an ectopic cell are found in both a wild type (**B**) and a *tm5268* mutant (**D**). A PVD neuron shows characteristic branching dendrites in both N2 (**E**) and *tm5268* (**F**) worms. Insets show Norarski images of the same areas. **G–J**: Expression patterns of DAT-1p::GFP in wild-type (n = 10) and *tm5268* (n = 15) worms carrying the *dat-1p::gfp* reporter (*baIs4*). Nomarski (**G, I)** and GFP (**H, J**) images of wild-type (**G, H**) and *tm5268* (**I, J**) adult hermaphrodites. White arrows indicate PDEs. **K–N**: Patterns of absorbance of fluorescent dye in dye-filling assays. Nomarski (**K, M)** and DiI (**L, N**) images of wild-type (**K, L**) (n = 10) and *tm5268* (**M, N**) (n = 10) adult hermaphrodites. Arrowheads indicate a pair of socket cells in the phasmid. **O–R**: Expression patterns of MEC-4p::GFP in wild-type (n = 10) and *tm5268* (n = 12) worms carrying the *mec-4p::gfp* reporter (*bzIs8*). Nomarski (**O, Q)** and GFP (**P, R**) images of wild-type (**O, P**) and *tm5268* (**Q, R**) adult hermaphrodites. Asterisks indicate PLMs. **S, T**: The lineages of V1**–**V6 cells and T cells in wild-type hermaphrodites (Sulston & Horvitz, 1977). The directions of the cell divisions are shown with the anterior to the left and the posterior to the right. PHso1 and PHso2 are socket cells that support phasmid sensory neurons. PDE, PVD, PVN, PVW, PHC, and PLN are neurons. Circles indicate hyp7 cells, double circles indicate adult seam cells, and x indicates programmed cell death. Scale bar  = 50 µm.
(TIFF)

S5 Fig Knockout of *bcl-7* induces nuclear enlargement of epidermal cells. **A, B**: Examples of GFP localization in hyp7 cells of wild-type (**A**) and *tm5268* (**B**) hermaphrodites carrying the *col-19::gfp* reporter. **C, D**: Histograms of the length of the major axis of hyp7 cell nuclei in wild-type (**C**) and *tm5268* (**D**) hermaphrodites. Counted cells of wild-type and *tm5268* were more than 300. Scale bar  = 10 µm.
(TIFF)

S6 Fig BCL-7 affects cell differentiation in *C. elegans*. **A–H**: Nomarski (A, C, E, G) and RFP (B, D, F, H) images of wild-type (n = 10) and *tm5268* (n = 16) L4-stage hermaphrodites carrying the *egl-27p::HIS-24::mCherry* reporter (*stIs10165*). The exposure time was 800 msec for mCherry pictures. A wild-type hermaphrodite expressing EGL-27p::HIS-24::mCherry strongly in intestinal cells (white arrowheads indicate a part of the intestinal cells expressing mCherry) and weakly in hypodermis (“s” indicates a seam cell; “h” indicates a hyp7 cell) (**B, D**) and a *tm5268* hermaphrodite expressing mCherry strongly in seam cells and hyp7 cells (**F, H**). **I**: mRNA expression of *egl-27* as assessed by qRT-PCR analysis. **J**: mRNA expression of *ceh-6* as assessed by qRT-PCR analysis. All experiments were performed more than three times independently. The mRNA expression levels of *tm5268* mutants were normalized by that of wild type worms. Error bars indicate SEM. The asterisks indicate the statistical significance of the differences between groups. **p<0.005, ***p<0.001. Scale bar  = 50 µm.
(TIFF)

S7 Fig Knockout of *bcl-7* affects the normal development of germ cells. **A–D**: Histograms of the length of the major axis of germ cell nuclei in wild-type (**A**), *tm5268* (**B**), *tm5268;tmEx2966* carrying the *Pbcl-7::bcl-7::egfp* reporter as a rescue construct (**C**), and *tm5268;tmEx3873* carrying the *Plag-2::bcl-7::egfp* reporter as a DTC-specific rescue construct (**D**) adult hermaphrodites. **E**: A graph showing the percentages of Ste phenotypes in adult hermaphrodites of *bcl-7* worms with *Pbcl-7::bcl-7::KillerRed* (*tm5268;tmEx3878*) with or without green light illumination. Thick black lines indicate the exposed periods. A black circle indicates worms without exposure. Figs. in parentheses indicate the number of treated worms.
(TIFF)

S8 Fig Knockout of *bcl-7* inhibits normal differentiation of distal tip cells (DTCs) in *Caenorhabditis elegans*. **A–H**: Nomarski (**A, C, E, G**) and GFP (**B, D, F, H)** images of wild-type (**A, B**) and *tm5268* (**C–F**) adult hermaphrodites carrying a *lag-2p::gfp* reporter (*qIs56*). A higher magnification view of the white square is presented in the lower panel of the images (**G, H**). **I–N**: Nomarski (**I, K, M**) and GFP (**J, L, N)** images of wild-type (**I, J**) and *tm5268* (**K–N**) L3-stage hermaphrodites carrying the *hlh-2p::gfp::hlh-2* reporter (*qyIs174*). A DTC is outlined with dotted lines (**I–N**). Arrowheads indicate GFP expression in DTCs (**B, D, F, H, J, N**). Asterisks indicate mispositioning of DTCs with Nomarski images and GFP expression (**F–H, M, N**). VC, vulval cell (**B, L**). Scale bar  = 50 µm.
(TIFF)

S9 Fig Knockout of *bcl-7* affects several pathways in *C. elegans*. **A**: mRNA expression of *bar-1*, *wrm-1*, and *sys-1*, *C. elegans* homologues of human *ß-catenin*, as assessed by qRT-PCR analysis. **B**. mRNA expression of *ced-9*, a *C. elegans* homolog of a human anti-apoptotic factor, *Bcl2*, as assessed by qRT-PCR analysis. All experiments were performed more than three times independently. Each data was normalized to the expression level of wild type worms. Error bars indicate SEM. The asterisks indicate the statistical significance of the differences between groups. *p<0.05, ***p<0.001.
(TIFF)

S10 Fig *BCL7B* downregulation shows various phenotypes but not aneuploidy. **A, B**: Histograms of the area of nuclei in KATOIII cells transfected with control-siRNA (**A**) or *BCL7B*-siRNA (**B**). **C, D**: Cell cycle profiles of cells transfected with control-siRNA (**C**) or *BCL7B*-siRNA (**D**). **E**: Percentages and numbers of KATOIII cells present in each stage of the cell cycle. **F, G**: mRNA expression of *BCL7B* (**F**) and *HMGA1* (**G**), as assessed by qRT-PCR analysis. The experiments were performed three times independently. The relative mRNA level of *BCL7B*-downregulated KATOIII cells were normalized by that of the control. Error bars indicate SEM. The asterisks indicate the statistical significance of the differences between groups. *p<0.05, ***p<0.001.
(TIFF)

S11 Fig Line graphs of relative mRNA levels in KATOIII cells. **A**: mRNA expression of *Oct4*, *Nanog*, and *Sox2*, as assessed by qRT-PCR analaysis. **B**: mRNA expression of *PTEN*, *Bax*, and *Bcl2*, as assessed by qRT-PCR analysis. All experiments were performed three times independently. The relative mRNA levels of *BCL7B*-knockdown KATOIII cells were normalized by that of the control. Error bars indicate SEM. The asterisks indicate the statistical significance of the differences between groups. *p<0.05, **p<0.005, ***p<0.001.
(TIFF)

S12 Fig RNA is increased in the KATOIII cells with the downregulation of *BCL7B*. **A–H**: KATOIII cells stained with DAPI (**A–D**) or ethidium bromide (**E–H**) with (**C, D, G, H**) or without RNase (**A, B, E, F**). **I**: A line graph of the ratio of the fluorescence intensity in KATOIII cells transfected with control-siRNA or *BCL7B-*siRNA stained with DAPI or ethidium bromide (EtBr). More than one hundred cells were counted. **J**: A line graph of the RNA expression of *NEAT1*, as analyzed by qRT-PCR. The experiments were performed three times independently. Error bars indicate SEM. The asterisks indicate the statistical significance of the differences between groups. *p<0.05, **p<0.005. N.S.: no significance. Scale bar  = 25 µm.
(TIFF)

S13 Fig Overexpression of *BCL7B* may regulate the apoptotic pathway positively. **A**: Total cell count of cells transfected with *pEGFP_N3* (black line) as a control or *BCL7B_EGFP* (red line). **B, C**: Example data of apoptosis assays. KATOIII cells transfected with *pEGFP_N3* (**B**) or *BCL7B_EGFP* (**C**) were stained with Annexin V (AV)/7-AAD (7A) and analyzed using a flow cytometer. More than ten thousand cells were counted, and these experiments were repeated three times independently. The AV^+^/7A^-^ population is shown in the R2 region of the left upper quadrant and represents early-apoptotic cells. The AV^+^/7A^+^ population is shown in the R3 region of the right upper quadrant and represents late-apoptotic cells. The gating lines were drawn based on the viable cells as the negative control, which belongs to the left lower quadrant. Error bars indicate SEM.
(TIFF)

S14 Fig A schematic model of the functions of BCL-7/BCL7B. BCL7B and its *C. elegans* homologous protein, BCL-7, may function as a tumor suppressor through multiple pathways, including the apoptosis and the Wnt signaling pathway, which inhibits and promotes cancer, respectively. BCL-7 and BCL7B also promote terminal cell differentiation, which is important for cancer malignancy.
(TIFF)

S1 Table Number of DTC expressing *lag-2p::gfp* in wild-type worms or *bcl-7* mutants. "mispositioning" means worms with GFP-positive mispositioned DTC with or without normal positioning of DTC (such as S8 Fig.E-H). All examined animals were mounted on slide-glasses and observed using a fluorescence microscope.
(DOC)

S2 Table Lists of genes for the RNAi screening.
(XLS)

S3 Table Average number of GFP-positive seam cells in RNAi experiments. "*wrm-1c* RNAi" means RNAi experiments using with *wrm-1* RNAi clone constructed with a cDNA of wild-type worms. “1/10 *wrm-1c* RNAi” means diluted RNAi as described in the text.
(DOC)

S4 Table Number of DTCs expressing GFP::HLH-2 in wild-type worms or *bcl-7* mutants. "mispositioning" means worms with GFP-positive mispositioned DTC with or without normal positioning of DTC (such as S8M Fig. and S8N Fig.). All examined animals were mounted on slide-glasses and observed using a fluorescence microscope.
(DOC)

S5 Table List of primers used for producing RNAi clones.
(XLS)

S6 Table List of primers used for the qRT-PCR experiments in *C. elegans*.
(XLS)

S7 Table List of primers used for the qRT-PCR experiments in KATOIII cells.
(XLS)
