# Supplementary figures and images for: The Tumor Suppressor BCL7B Functions in the Wnt Signaling Pathway
Source: PLoS Genet. 2015 Jan 8;11(1):e1004921. doi: 10.1371/journal.pgen.1004921 (PMC4287490; doi:10.1371/journal.pgen.1004921)

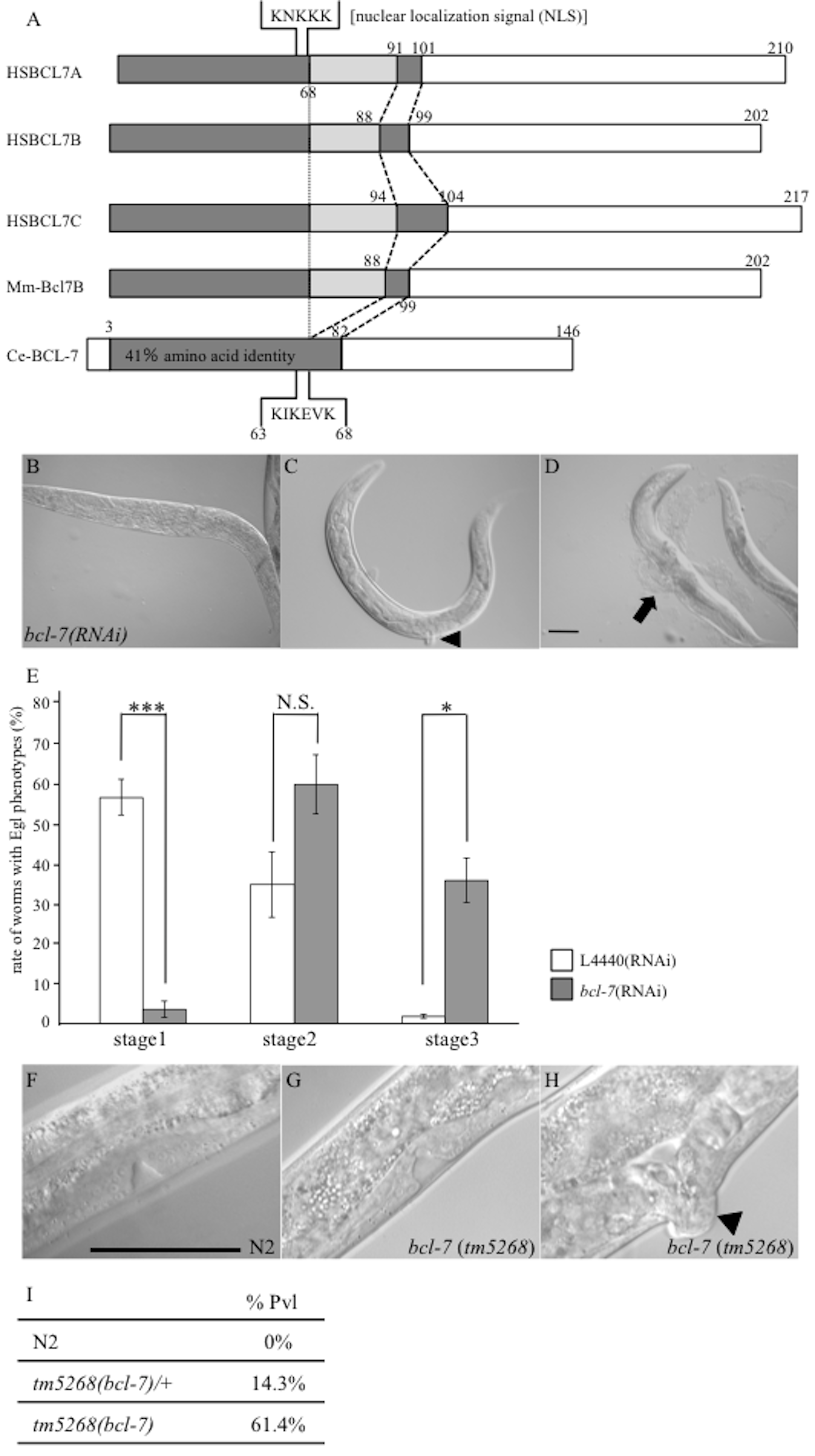

Supplement: S1 Fig — Decrease or loss of BCL-7 results in a variety of phenotypes, including Egl, Pvl, and Burst in Caenorhabditis elegans. A: A schematic diagram of protein homology of human BCL7 family (hsBCL7A, hsBCL7B, and hsBCL7C), Mus musculus homolog of BCL7B (Mm-Bcl7b), and C. elegans homolog (CeBcl-7) adapted from Ref.10. Numbers indicate amino acid positions; gray boxes indicate high homologous sequences; white boxes indicate the regions that are not conserved in C. elegans. B–D: Nomarski images of adult hermaphrodites treated with bcl-7-RNAi. Arrowheads indicate the Pvl phenotype. An arrow indicates the Burst phenotype. E: A bar chart representing the frequency of worms with the Egl phenotype (n = 50–70, and these experiments were repeated five times independently). Egg-laying behavior was assayed as follows. The developmental stage of eggs inside the uteri of the worms, determined by microscopy, was categorized as the 1- to 8-cell stage (stage 1; 1); 16-cell stage to precomma stage (stage 2; 2); or comma to postcomma stage (stage 3; 3). F–H: Nomarski images of a wild-type (F) and tm5268 hermaphrodites (G) at the L4 stage and at the adult-stage (H). I: Percentages of the Pvl phenotypes in wild type and tm5268 mutant worms (n>80). Error bars indicate SEM. Asterisks indicate the statistical significance of differences between groups. *p<0.05. ***p<0.001. N.S.: no significance. Scale bar = 50 µm. (TIFF) [file pgen.1004921.s001.tiff]

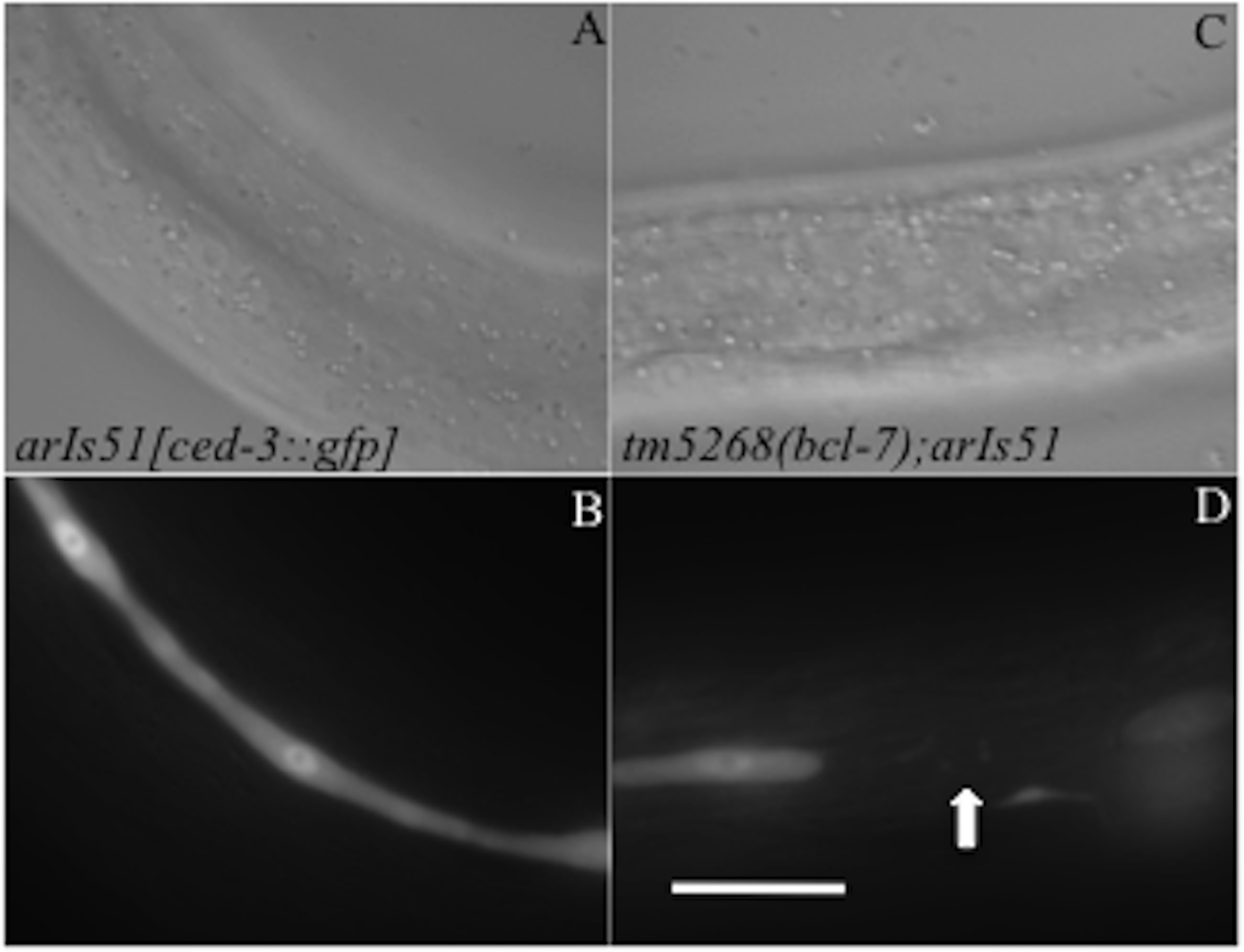

Supplement: S2 Fig — Knockout of bcl-7 inhibits normal epidermal development in Caenorhabditis elegans. A–D: Examples of GFP localization in wild-type and tm5268 hermaphrodites carrying the cdh-3::gfp reporter. Nomarski (A, C) and GFP (B, D) images of wild-type (A, B) and tm5268 (C, D) adult hermaphrodites carrying the cdh-3::gfp reporter. A white arrow indicates the absence of a seam cell. Scale bar = 25 µm. (TIFF) [file pgen.1004921.s002.tiff]

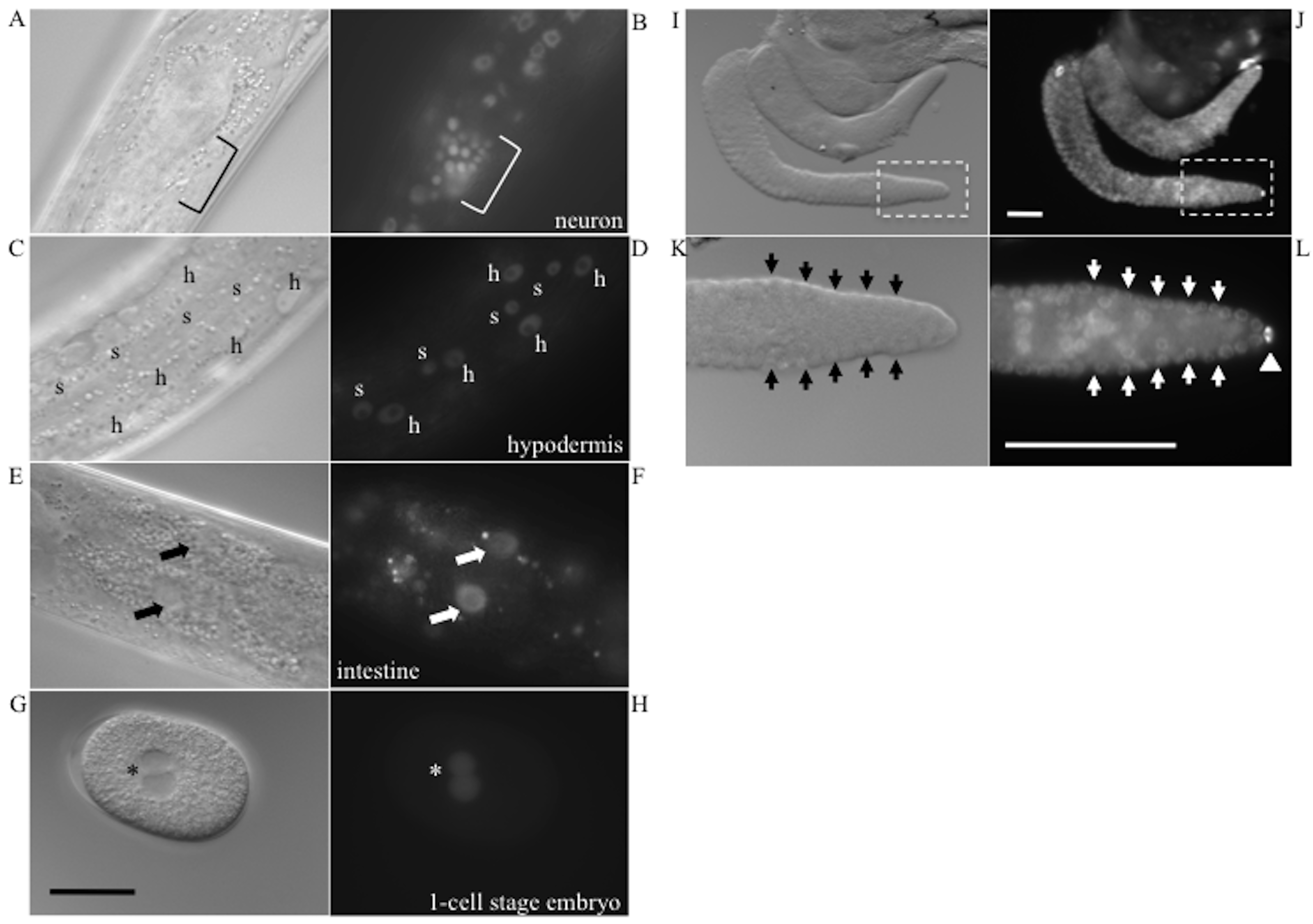

Supplement: S3 Fig — BCL-7 is ubiquitously expressed. A–L: Nomarski (A, C, E, G, L, K) and GFP (B, D, F, H, J, L) images of wild-type adult hermaphrodites carrying the Pbcl-7::bcl-7::egfp reporter. BCL-7 is expressed in the nuclei of neurons (bracket) (B), the seam cells (‘s’) and hyp7 cells (‘h’) (D), and intestines (white arrows) (F) of worms. BCL-7 is expressed in the early embryonic stage (asterisk) (H). BCL-7 was expressed in germ cells and is strongly expressed in a somatic distal tip cell (DTC) (J, L). A higher magnification view of the white square is presented in the lower panel of the images (K, L). The arrowhead indicates strong GFP expression in the DTC. The white arrows indicate GFP expression in gonadal sheath cells. Scale bar = 50 µm. (TIFF) [file pgen.1004921.s003.tiff]

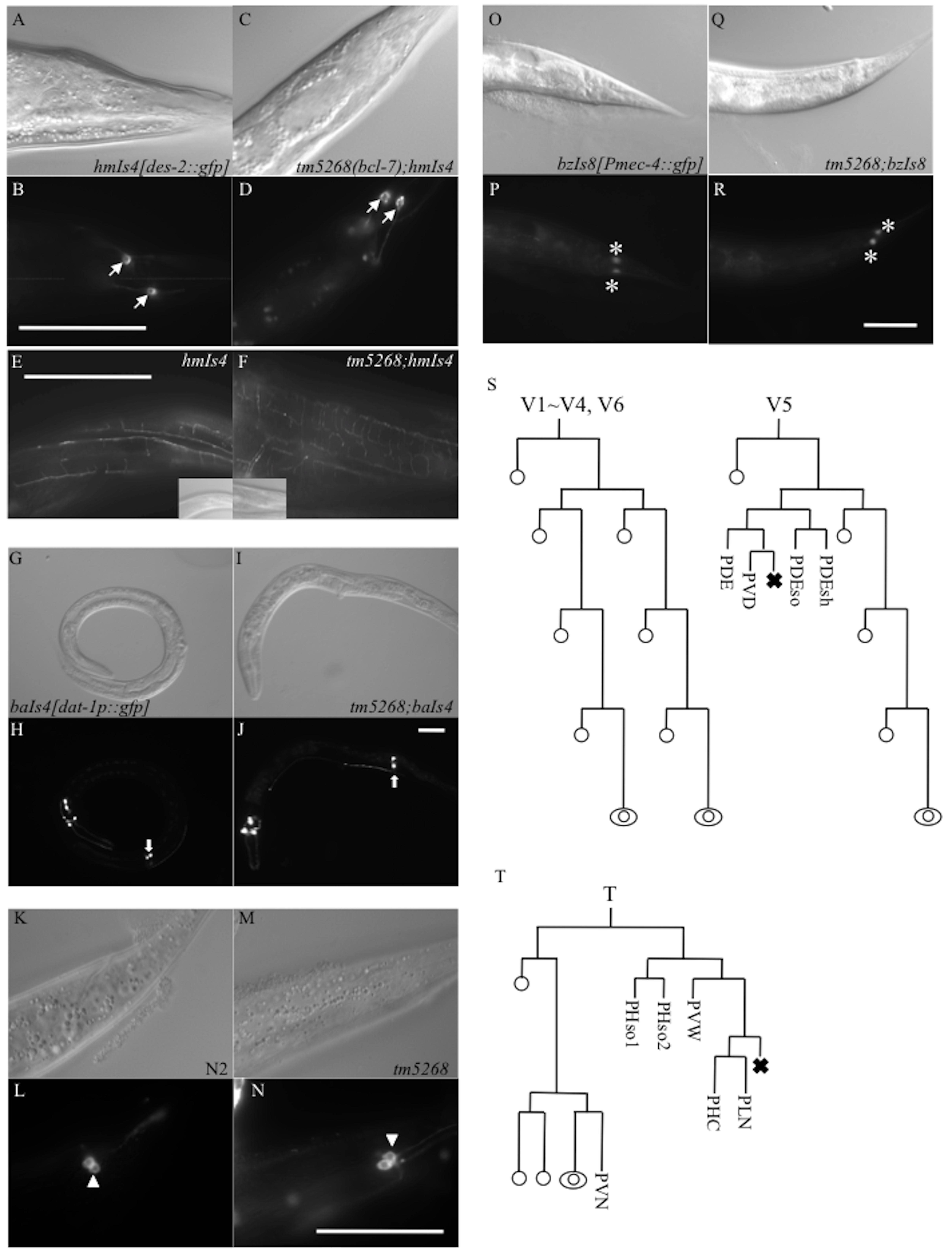

Supplement: S4 Fig — Knockout of bcl-7 has no effect on the development of neuronal cells. A–F: Expression patterns of DES-2::GFP in wild-type (n = 10) and tm5268 (n = 12) hermaphrodites carrying the des-2::gfp reporter (hmIs4). Nomarski (A, C) and GFP (B, D, E, F) images of wild-type (A, B, E) and tm5268 (C, D, F) adult hermaphrodites. Two PVD neurons (white arrows) without an ectopic cell are found in both a wild type (B) and a tm5268 mutant (D). A PVD neuron shows characteristic branching dendrites in both N2 (E) and tm5268 (F) worms. Insets show Norarski images of the same areas. G–J: Expression patterns of DAT-1p::GFP in wild-type (n = 10) and tm5268 (n = 15) worms carrying the dat-1p::gfp reporter (baIs4). Nomarski (G, I) and GFP (H, J) images of wild-type (G, H) and tm5268 (I, J) adult hermaphrodites. White arrows indicate PDEs. K–N: Patterns of absorbance of fluorescent dye in dye-filling assays. Nomarski (K, M) and DiI (L, N) images of wild-type (K, L) (n = 10) and tm5268 (M, N) (n = 10) adult hermaphrodites. Arrowheads indicate a pair of socket cells in the phasmid. O–R: Expression patterns of MEC-4p::GFP in wild-type (n = 10) and tm5268 (n = 12) worms carrying the mec-4p::gfp reporter (bzIs8). Nomarski (O, Q) and GFP (P, R) images of wild-type (O, P) and tm5268 (Q, R) adult hermaphrodites. Asterisks indicate PLMs. S, T: The lineages of V1–V6 cells and T cells in wild-type hermaphrodites (Sulston & Horvitz, 1977). The directions of the cell divisions are shown with the anterior to the left and the posterior to the right. PHso1 and PHso2 are socket cells that support phasmid sensory neurons. PDE, PVD, PVN, PVW, PHC, and PLN are neurons. Circles indicate hyp7 cells, double circles indicate adult seam cells, and x indicates programmed cell death. Scale bar = 50 µm. (TIFF) [file pgen.1004921.s004.tiff]

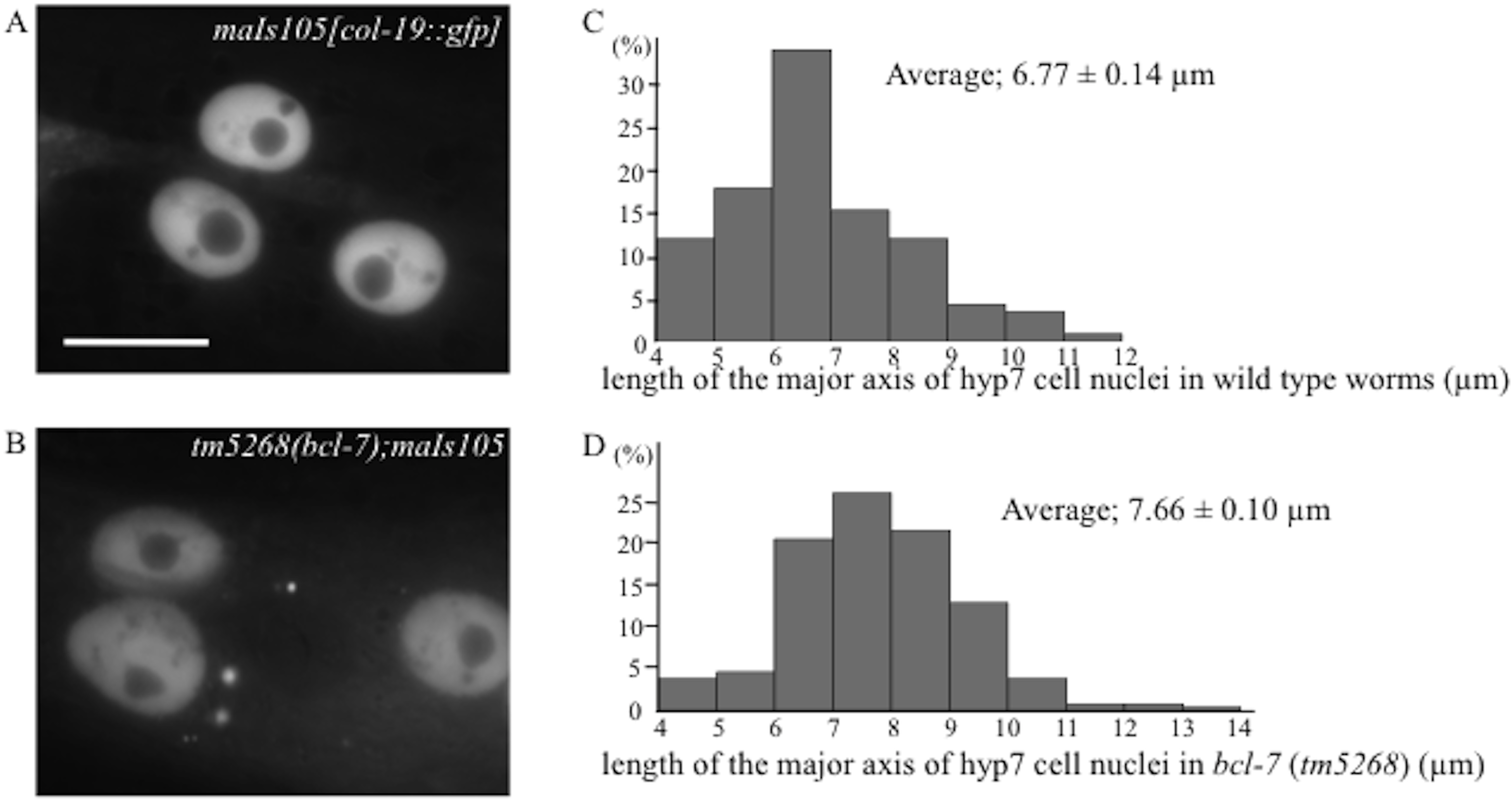

Supplement: S5 Fig — Knockout of bcl-7 induces nuclear enlargement of epidermal cells. A, B: Examples of GFP localization in hyp7 cells of wild-type (A) and tm5268 (B) hermaphrodites carrying the col-19::gfp reporter. C, D: Histograms of the length of the major axis of hyp7 cell nuclei in wild-type (C) and tm5268 (D) hermaphrodites. Counted cells of wild-type and tm5268 were more than 300. Scale bar = 10 µm. (TIFF) [file pgen.1004921.s005.tiff]

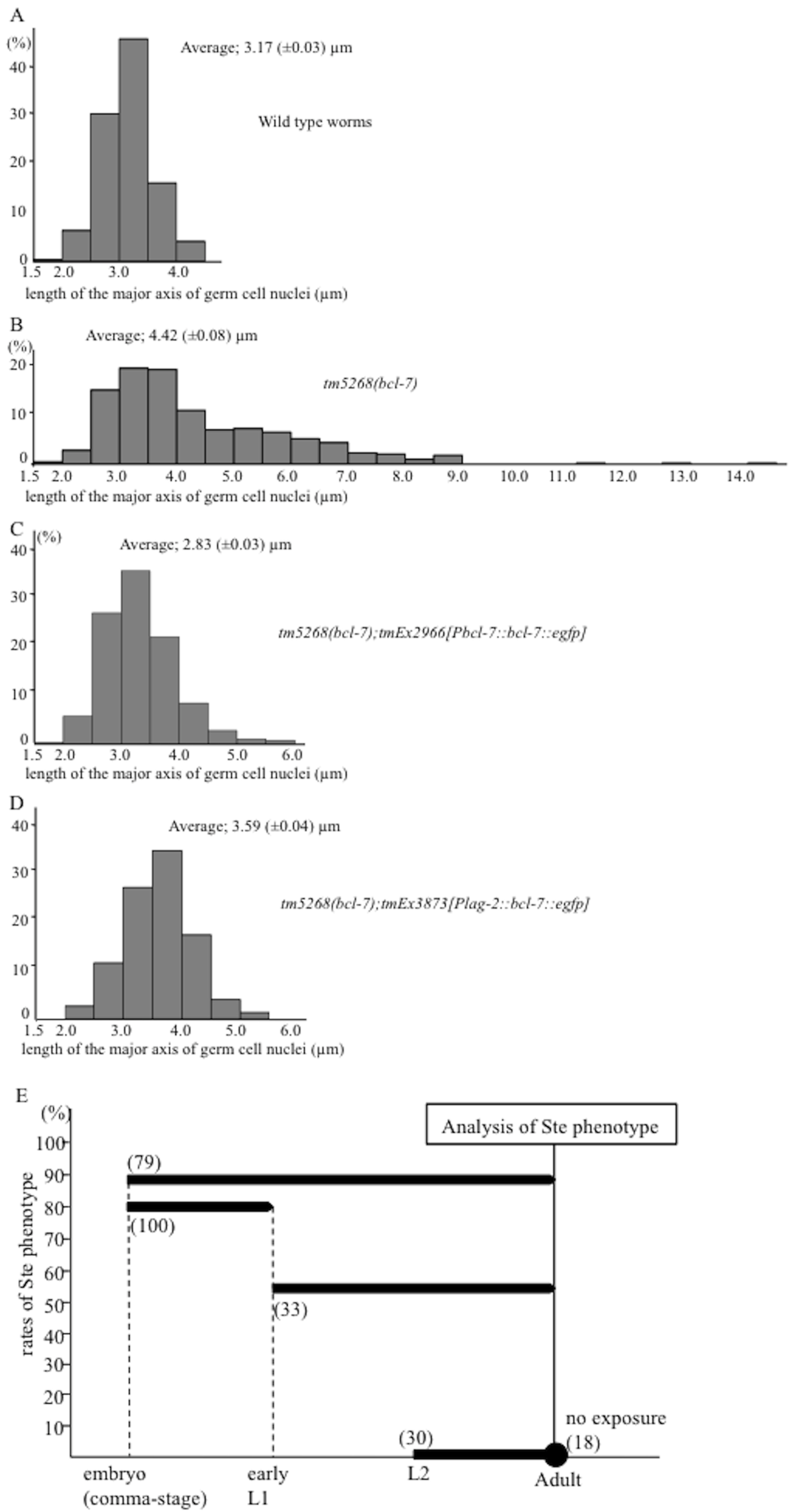

Supplement: S7 Fig — Knockout of bcl-7 affects the normal development of germ cells. A–D: Histograms of the length of the major axis of germ cell nuclei in wild-type (A), tm5268 (B), tm5268;tmEx2966 carrying the Pbcl-7::bcl-7::egfp reporter as a rescue construct (C), and tm5268;tmEx3873 carrying the Plag-2::bcl-7::egfp reporter as a DTC-specific rescue construct (D) adult hermaphrodites. E: A graph showing the percentages of Ste phenotypes in adult hermaphrodites of bcl-7 worms with Pbcl-7::bcl-7::KillerRed (tm5268;tmEx3878) with or without green light illumination. Thick black lines indicate the exposed periods. A black circle indicates worms without exposure. Figs. in parentheses indicate the number of treated worms. (TIFF) [file pgen.1004921.s007.tiff]

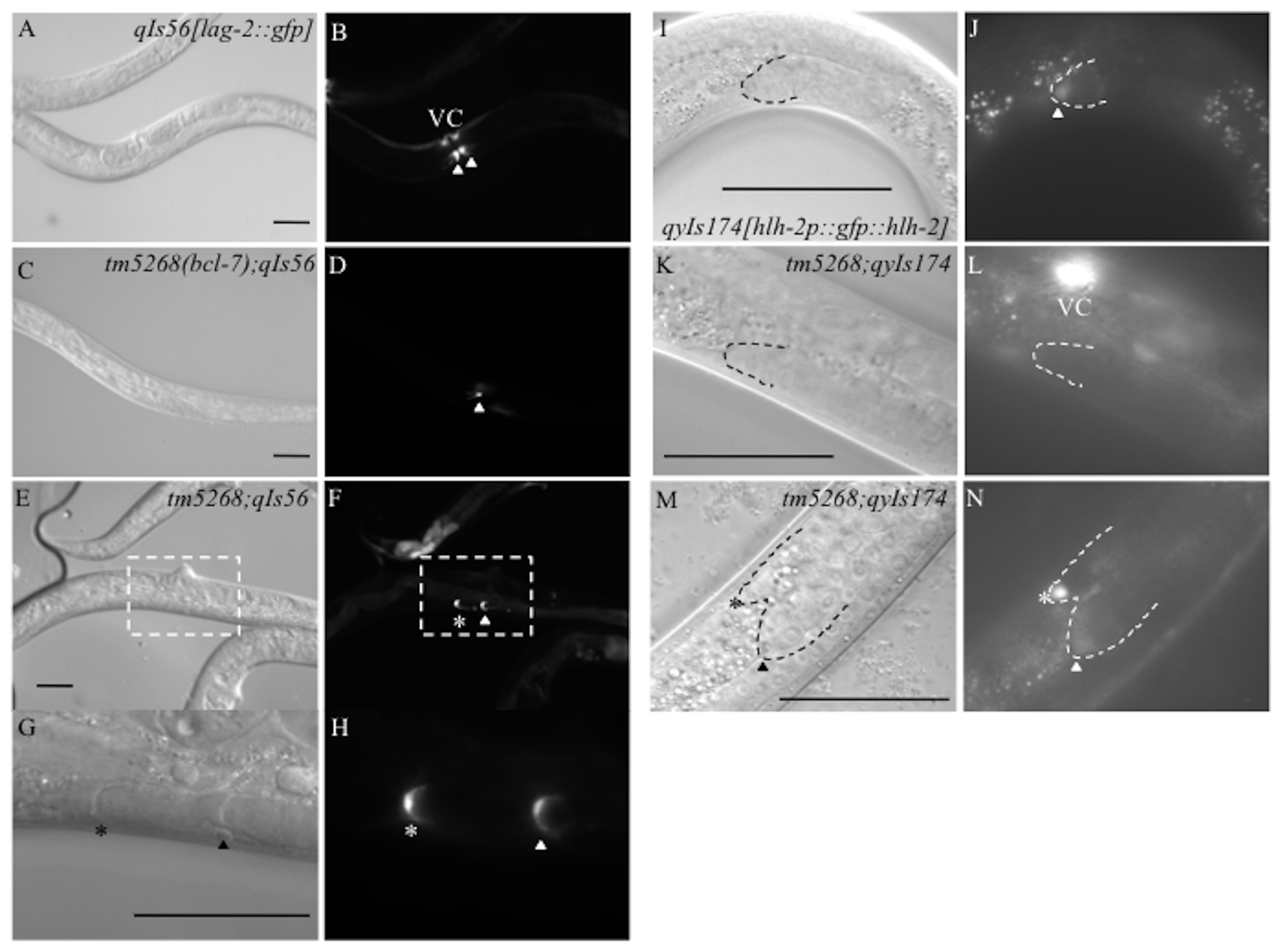

Supplement: S8 Fig — Knockout of bcl-7 inhibits normal differentiation of distal tip cells (DTCs) in Caenorhabditis elegans. A–H: Nomarski (A, C, E, G) and GFP (B, D, F, H) images of wild-type (A, B) and tm5268 (C–F) adult hermaphrodites carrying a lag-2p::gfp reporter (qIs56). A higher magnification view of the white square is presented in the lower panel of the images (G, H). I–N: Nomarski (I, K, M) and GFP (J, L, N) images of wild-type (I, J) and tm5268 (K–N) L3-stage hermaphrodites carrying the hlh-2p::gfp::hlh-2 reporter (qyIs174). A DTC is outlined with dotted lines (I–N). Arrowheads indicate GFP expression in DTCs (B, D, F, H, J, N). Asterisks indicate mispositioning of DTCs with Nomarski images and GFP expression (F–H, M, N). VC, vulval cell (B, L). Scale bar = 50 µm. (TIFF) [file pgen.1004921.s008.tiff]

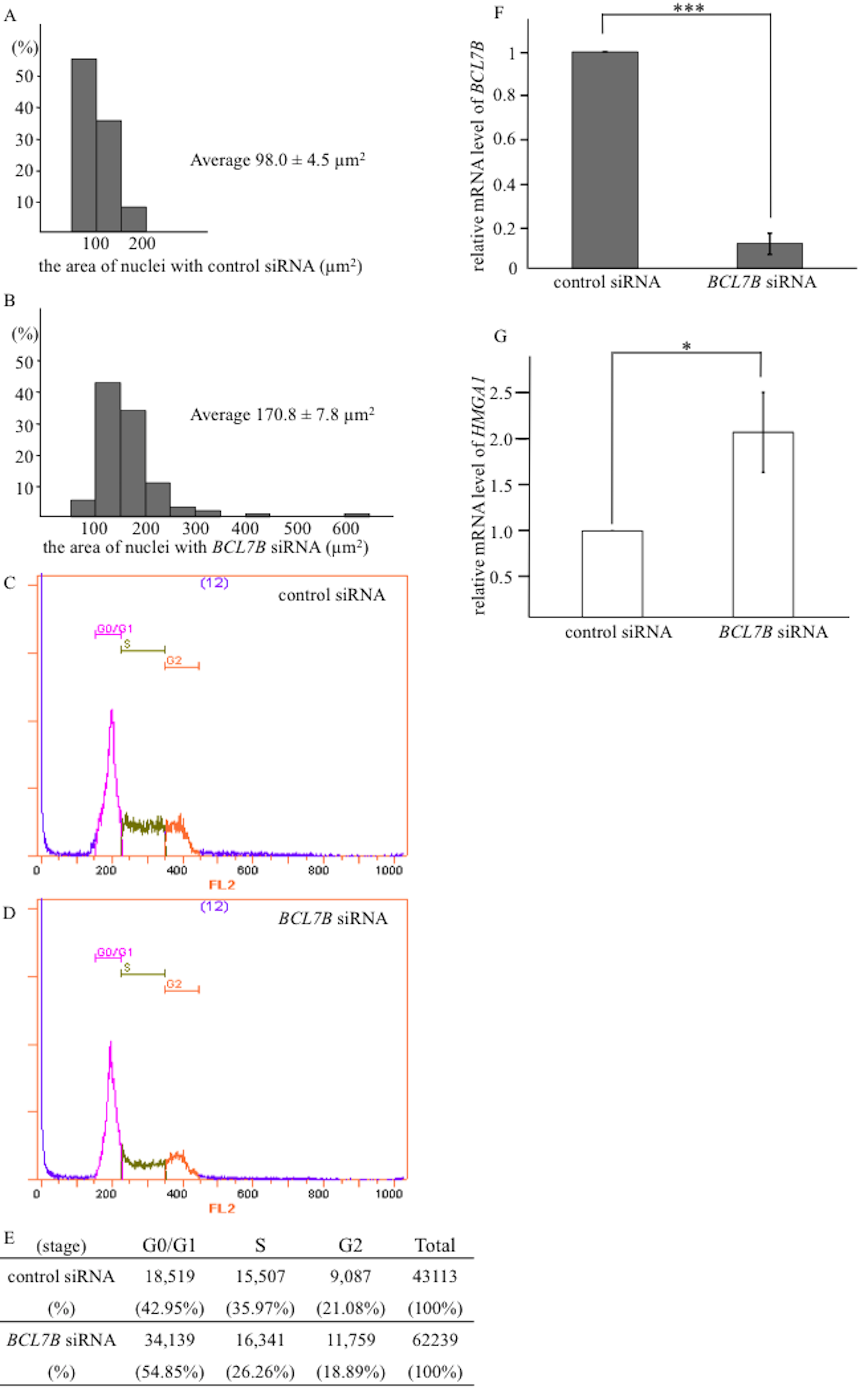

Supplement: S10 Fig — BCL7B downregulation shows various phenotypes but not aneuploidy. A, B: Histograms of the area of nuclei in KATOIII cells transfected with control-siRNA (A) or BCL7B-siRNA (B). C, D: Cell cycle profiles of cells transfected with control-siRNA (C) or BCL7B-siRNA (D). E: Percentages and numbers of KATOIII cells present in each stage of the cell cycle. F, G: mRNA expression of BCL7B (F) and HMGA1 (G), as assessed by qRT-PCR analysis. The experiments were performed three times independently. The relative mRNA level of BCL7B-downregulated KATOIII cells were normalized by that of the control. Error bars indicate SEM. The asterisks indicate the statistical significance of the differences between groups. *p<0.05, ***p<0.001. (TIFF) [file pgen.1004921.s010.tiff]

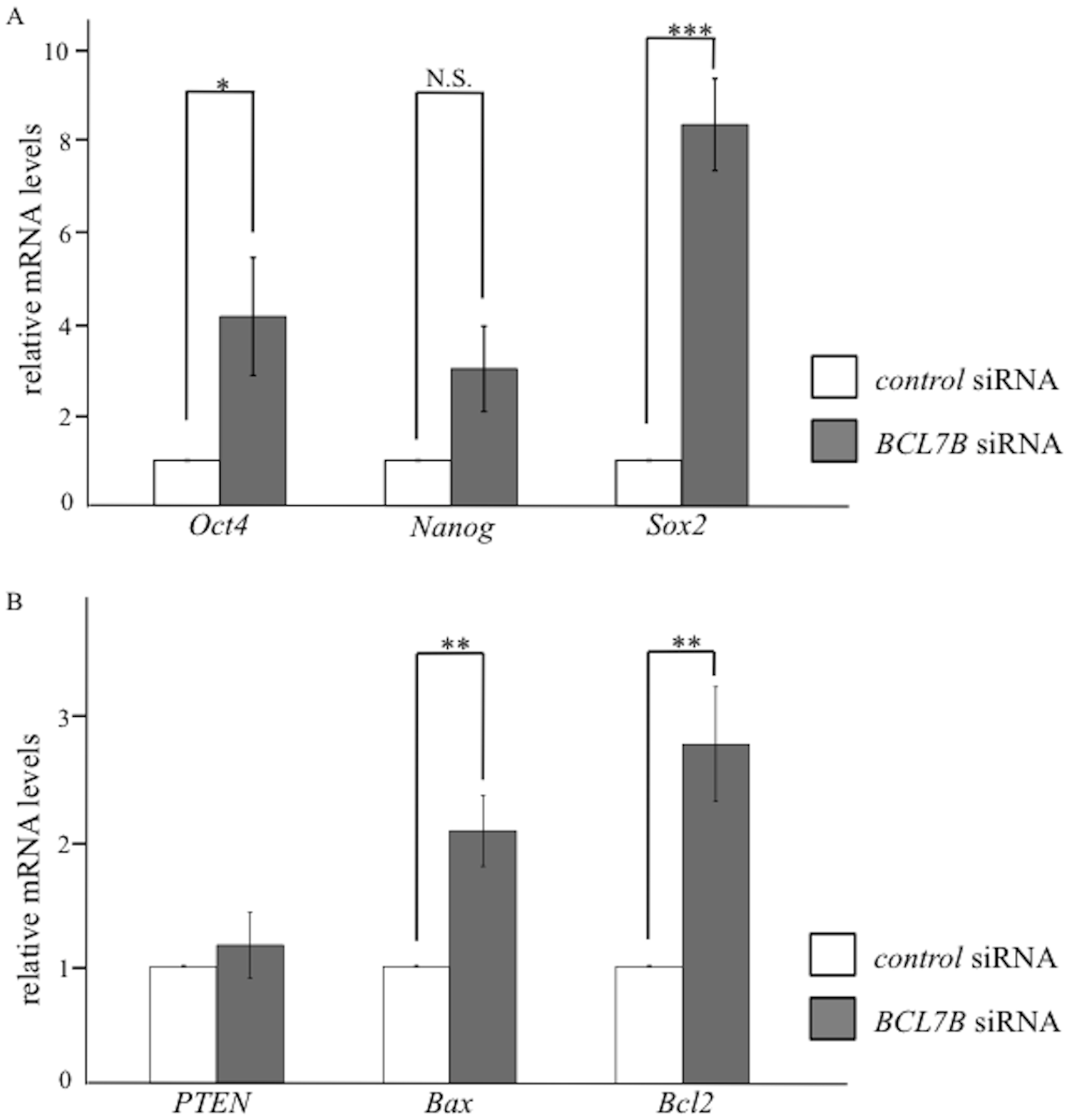

Supplement: S11 Fig — Line graphs of relative mRNA levels in KATOIII cells. A: mRNA expression of Oct4, Nanog, and Sox2, as assessed by qRT-PCR analaysis. B: mRNA expression of PTEN, Bax, and Bcl2, as assessed by qRT-PCR analysis. All experiments were performed three times independently. The relative mRNA levels of BCL7B-knockdown KATOIII cells were normalized by that of the control. Error bars indicate SEM. The asterisks indicate the statistical significance of the differences between groups. *p<0.05, **p<0.005, ***p<0.001. (TIFF) [file pgen.1004921.s011.tiff]

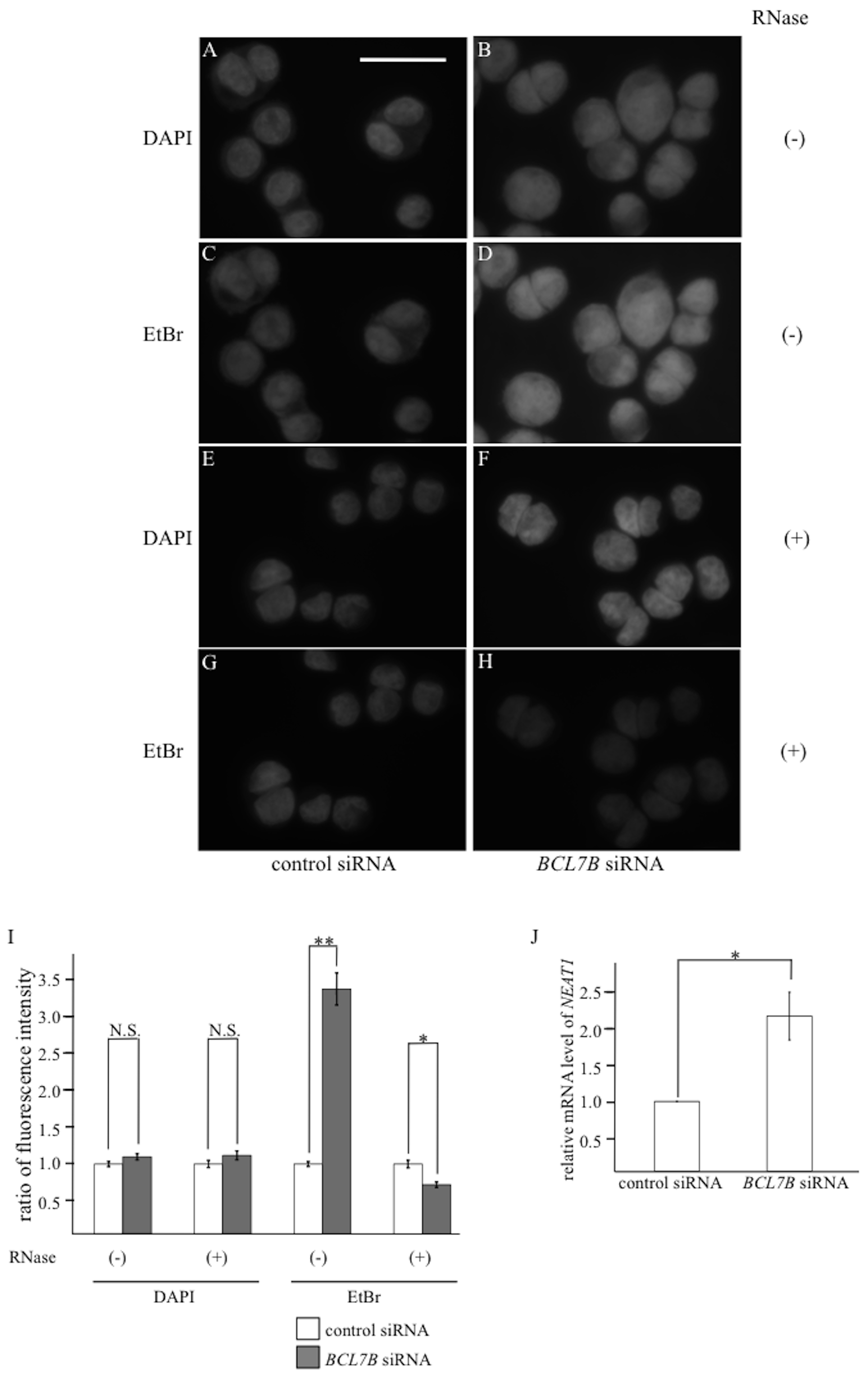

Supplement: S12 Fig — RNA is increased in the KATOIII cells with the downregulation of BCL7B. A–H: KATOIII cells stained with DAPI (A–D) or ethidium bromide (E–H) with (C, D, G, H) or without RNase (A, B, E, F). I: A line graph of the ratio of the fluorescence intensity in KATOIII cells transfected with control-siRNA or BCL7B-siRNA stained with DAPI or ethidium bromide (EtBr). More than one hundred cells were counted. J: A line graph of the RNA expression of NEAT1, as analyzed by qRT-PCR. The experiments were performed three times independently. Error bars indicate SEM. The asterisks indicate the statistical significance of the differences between groups. *p<0.05, **p<0.005. N.S.: no significance. Scale bar = 25 µm. (TIFF) [file pgen.1004921.s012.tiff]

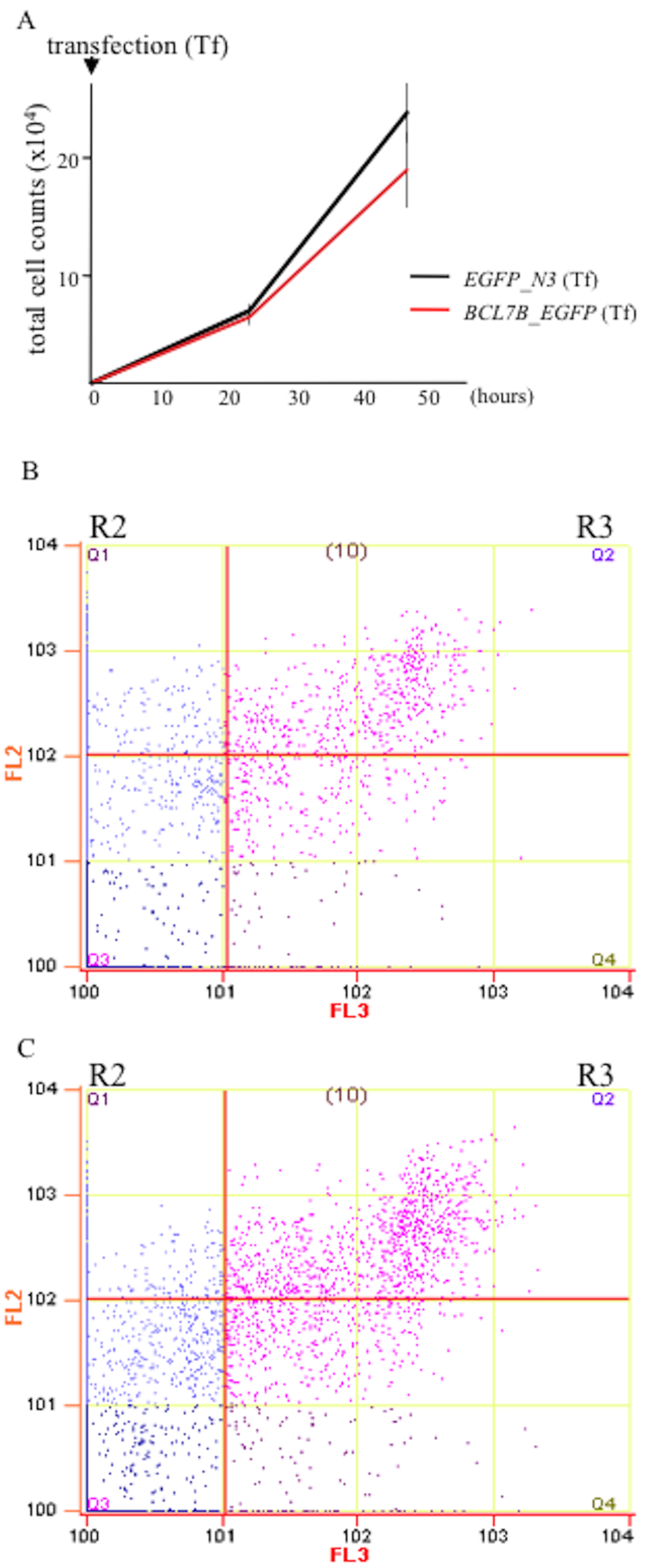

Supplement: S13 Fig — Overexpression of BCL7B may regulate the apoptotic pathway positively. A: Total cell count of cells transfected with pEGFP_N3 (black line) as a control or BCL7B_EGFP (red line). B, C: Example data of apoptosis assays. KATOIII cells transfected with pEGFP_N3 (B) or BCL7B_EGFP (C) were stained with Annexin V (AV)/7-AAD (7A) and analyzed using a flow cytometer. More than ten thousand cells were counted, and these experiments were repeated three times independently. The AV+/7A- population is shown in the R2 region of the left upper quadrant and represents early-apoptotic cells. The AV+/7A+ population is shown in the R3 region of the right upper quadrant and represents late-apoptotic cells. The gating lines were drawn based on the viable cells as the negative control, which belongs to the left lower quadrant. Error bars indicate SEM. (TIFF) [file pgen.1004921.s013.tiff]

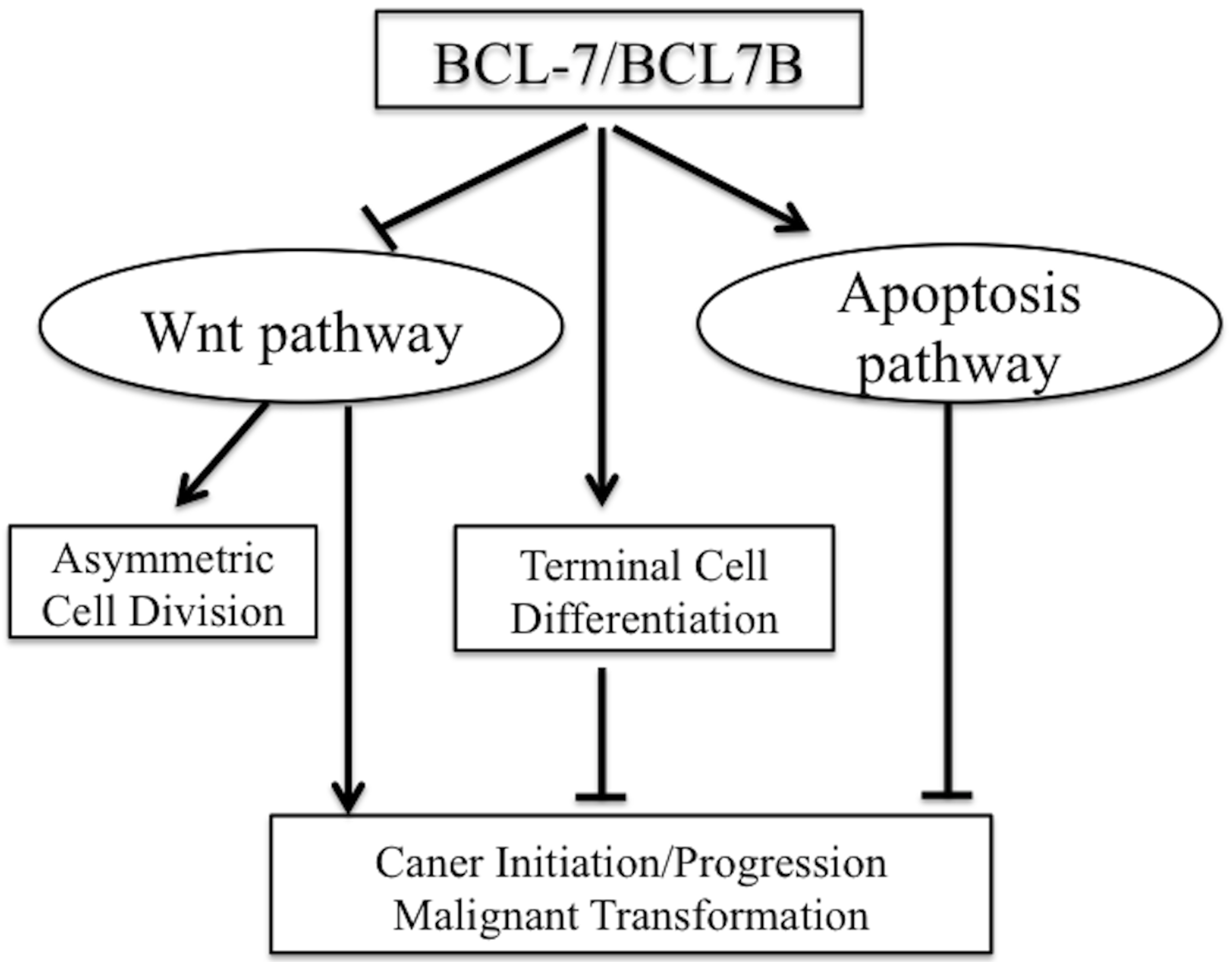

Supplement: S14 Fig — A schematic model of the functions of BCL-7/BCL7B. BCL7B and its C. elegans homologous protein, BCL-7, may function as a tumor suppressor through multiple pathways, including the apoptosis and the Wnt signaling pathway, which inhibits and promotes cancer, respectively. BCL-7 and BCL7B also promote terminal cell differentiation, which is important for cancer malignancy. (TIFF) [file pgen.1004921.s014.tiff]
