# Supplementary material for: The Tumor Suppressor BCL7B Functions in the Wnt Signaling Pathway
Source: PLoS Genet. 2015 Jan 8;11(1):e1004921. doi: 10.1371/journal.pgen.1004921 (PMC4287490; doi:10.1371/journal.pgen.1004921)
Supplement: S1 Table — Number of DTC expressing lag-2p::gfp in wild-type worms or bcl-7 mutants. "mispositioning" means worms with GFP-positive mispositioned DTC with or without normal positioning of DTC (such as S8 Fig.E-H). All examined animals were mounted on slide-glasses and observed using a fluorescence microscope. (DOC) [file pgen.1004921.s015.doc]

Table S1. Number of DTC expressing *lag-2::gfp* in wild-type worms or *bcl-7* mutants.

"mispositioning" means worms with GFP-positive mispositioned DTC with or without normal positioning of DTC (such as Figure S8E-H).

All examined animals were mounted on slide-glasses and observed by using a fluorescent microscope.

| cell number | 2 | 1 | 0 | (mispositioning) |  |
| --- | --- | --- | --- | --- | --- |
| *qIs56* | 17 | 0 | 0 | (0) | n=17 |
| *tm5268/+;qIs56* | 14 | 6 | 0 | (10) | n=20 |
| *tm5268;qIs56* | 25 | 103 | 0 | (16) | n=128 |
