# Supplementary material for: The Tumor Suppressor BCL7B Functions in the Wnt Signaling Pathway
Source: PLoS Genet. 2015 Jan 8;11(1):e1004921. doi: 10.1371/journal.pgen.1004921 (PMC4287490; doi:10.1371/journal.pgen.1004921)
Supplement: S3 Table — Average number of GFP-positive seam cells in RNAi experiments. "wrm-1c RNAi" means RNAi experiments using with wrm-1 RNAi clone constructed with a cDNA of wild-type worms. “1/10 wrm-1c RNAi” means diluted RNAi as described in the text. (DOC) [file pgen.1004921.s017.doc]

Table S3. Average number of GFP-positive seam cells in RNAi experiments.

"*wrm-1c* RNAi" are RNAi experiments using with *wrm-1* RNAi clone constructed with a cDNAs of wild-type worms. “1/10 *wrm-1c* RNAi” means diluted RNAi as described in the text.

|  | control RNAi | *wrm-1c* RNAi | 1/10 *wrm-1c* RNAi |
| --- | --- | --- | --- |
| N2 | 16 | 1.33 ± 0.28 | 16 |
| *tm5268* | 2.83 ± 0.34 | 1.86 ± 0.40 | 8.08 ± 0.75 |
