# Supplementary material for: The Tumor Suppressor BCL7B Functions in the Wnt Signaling Pathway
Source: PLoS Genet. 2015 Jan 8;11(1):e1004921. doi: 10.1371/journal.pgen.1004921 (PMC4287490; doi:10.1371/journal.pgen.1004921)
Supplement: S4 Table — Number of DTCs expressing GFP::HLH-2 in wild-type worms or bcl-7 mutants. "mispositioning" means worms with GFP-positive mispositioned DTC with or without normal positioning of DTC (such as S8M Fig. and S8N Fig.). All examined animals were mounted on slide-glasses and observed using a fluorescence microscope. (DOC) [file pgen.1004921.s018.doc]

Table S4. Number of DTCs expressing *hlh-2p::gfp::hlh-2* in wild-type worms or *bcl-7* mutants.

"mispositioning" means worms with GFP-positive mispositioned DTC with or without normal positioning of DTC (such as Figure S8M and S8N).

All examined animals were mounted on slide-glasses and observed by using a fluorescent microscope.

| cell number | 2 | 1 | 0 | (mispositioning) |  |
| --- | --- | --- | --- | --- | --- |
| *qyIs174* | 10 | 0 | 0 | (0) | n=10 |
| *tm5268;qyIs174* | 13 | 28 | 5 | (21) | n=46 |
